# Normalization effect of levodopa on hierarchical brain function in Parkinson’s disease

**DOI:** 10.1162/netn_a_00232

**Published:** 2022-06-01

**Authors:** Tao Guo, Min Xuan, Cheng Zhou, Jingjing Wu, Ting Gao, Xueqin Bai, Xiaocao Liu, Luyan Gu, Ruiqi Liu, Zhe Song, Quanquan Gu, Peiyu Huang, Jiali Pu, Baorong Zhang, Xiaojun Xu, Xiaojun Guan, Minming Zhang

**Affiliations:** Department of Radiology, Second Affiliated Hospital, Zhejiang University School of Medicine, Hangzhou, China; Department of Neurology, Second Affiliated Hospital, Zhejiang University School of Medicine, Hangzhou, China; School of Information Science and Technology, ShanghaiTech University, Shanghai, China

**Keywords:** Parkinson’s disease, Graph theory, Diverse club, Rich club, Levodopa

## Abstract

Hierarchical brain organization, in which the rich club and diverse club situate in core position, is critical for global information integration in the human brain network. Parkinson’s disease (PD), a common movement disorder, has been conceptualized as a network disorder. Levodopa is an effective treatment for PD. Whether there is a functional divergence in the hierarchical brain system under PD pathology, and how this divergence is regulated by immediate levodopa therapy, remains unknown. We constructed a functional network in 61 PD patients and 89 normal controls and applied graph theoretical analyses to examine the neural mechanism of levodopa short response from the perspective of brain hierarchical configuration. The results revealed the following: (a) PD patients exhibited disrupted function within rich-club organization, while the diverse club preserved function, indicating a differentiated brain topological organization in PD. (b) Along the rich-club derivate hierarchical system, PD patients showed impaired network properties within rich-club and feeder subnetworks, and decreased nodal degree centrality in rich-club and feeder nodes, along with increased nodal degree in peripheral nodes, suggesting distinct functional patterns in different types of nodes. And (c) levodopa could normalize the abnormal network architecture of the rich-club system. This study provides evidence for levodopa effects on the hierarchical brain system with divergent functions.

## Author Summary

Many studies of brain networks have revealed densely connected regions forming the rich club and diverse club, which occupy the central position of the hierarchical brain system. Here, we explore the hierarchical topology in Parkinson’s disease (PD) and investigate the neural effect of levodopa on it. We show that within the core position of the hierarchical system, the function of the diverse club is preserved while the function of the rich club is impaired. Along the rich-club hierarchical system, the function of biologically costly rich-club and feeder subnetworks is disrupted, together with an increased function of peripheral nodes, which could be normalized by levodopa. Our study provides evidence of a disparity pattern between different levels of brain hierarchical systems under PD pathology.

## INTRODUCTION

Parkinson’s disease (PD) is a common neurodegenerative disorder characterized by hypodopaminergic neurotransmission within the nigrostriatal dopamine pathway (Braak et al., 2003; Lees, Hardy, & Revesz, 2009), leading to classic motor deficits (Kalia & Lang, 2015). Clinically, levodopa has become the most effective and widespread treatment for controlling PD symptoms (Cotzias, Van Woert, & Schiffer, 1967; Hauser, 2009). In recent years, network neuroscience approaches pointed to PD being a network-disconnection syndrome (Cronin-Golomb, 2010), and the network dysfunction could be represented by the functional abnormalities coupling various brain regions (Luo et al., 2015; Suo et al., 2017). In addition, some preliminary exploration had demonstrated the normalization effect of dopaminergic drugs on PD functional brain architecture (Ballarini et al., 2018; Berman et al., 2016). However, brain function is not solely attributable to the properties of individual regions but rather emerges from the network organization of the brain as a whole (Sporns, 2011). Specifically, the human brain is a hierarchical system in which different levels of the brain region jointly preserve overall brain function, but without much knowledge currently available. Therefore, although the dysfunction in PD connectome and the normalization effect of dopaminergic therapy on PD overall network measures have been indicated, whether there is a functional divergence between different levels of brain connectome under PD pathology, and how this divergence is regulated by immediate levodopa therapy, remains unknown.

To elucidate these questions, the first step was to characterize the brain hierarchical system. Network neuroscience revealed that there are a number of highly connected regions situated in the core position of the brain network (Hagmann et al., 2008). As indexed by a high degree centrality or high participation, these brain regions play a central role in overall network organization and have been identified as “brain hubs” (Sporns, Honey, & Kotter, 2007). Previous studies have demonstrated that some of these brain hub regions could generate a rich-club organization, in which these regions tend to be more densely connected among themselves than regions with a lower degree (van den Heuvel & Sporns, 2011). The rich club is functionally valuable for global neural signaling and interregional brain communication, and for providing information about the network’s hierarchical ordering (van den Heuvel, Kahn, Goni, & Sporns, 2012). Meanwhile, a group of brain regions with a high participation coefficient showing diverse connectivity are also strongly interconnected, forming the diverse club, which allows for integrating information and coordinating connectivity between communities, enabling local modular processing (Bertolero, Yeo, & D’Esposito, 2017). Based on these highly functionally connected organizations, the hierarchical brain system is depicted, which may contribute to the investigation of functional divergence of different brain levels in PD.

Next is how to transform these theories into practice. Neuroimaging analyses provide a powerful approach to map the brain network in vivo (Rubinov & Sporns, 2010). By employing resting-state functional magnetic resonance imaging (rs-fMRI), we could construct a large-scale functional network, wherein brain regions serve as nodes and the interregional functional connectivity represents edges. Combining the graph theory approaches, researchers have successfully addressed the hierarchical brain system in several disease statuses (R. Li et al., 2016; Stellmann et al., 2017; Verhelst, Vander Linden, De Pauw, Vingerhoets, & Caeyenberghs, 2018). This hierarchical brain model provides an avenue to investigate the functional divergence of different levels in the PD brain connectome, and by integrating with the immediate dopaminergic therapy, we could explore the neural effect of acute dopaminergic administration on various brain subsystems.

This study aimed to illustrate the neural mechanism of levodopa short response from the perspective of the brain hierarchical configuration. We hypothesized that PD patients would exhibit a disparity pattern between different levels of the brain hierarchical system and that immediate levodopa supplementation would exert a flexible modulation effect on them.

## MATERIALS AND METHODS

### Participants

All PD patients and normal controls signed informed consent forms in accordance with the approval of the Medical Ethics Committee of the Second Affiliated Hospital of Zhejiang University School of Medicine.

A total of 61 PD patients and 89 normal controls were included in this study. The diagnosis of PD was made by an experienced neurologist (B. Z.) according to the UK Parkinson’s Disease Society Brain Bank criteria (Hughes, Daniel, Kilford, & Lees, 1992). Normal controls and PD patients with a history of other neurologic or psychiatric disorders, brain trauma, or general exclusion criteria for MRI scanning were excluded from this study. Demographic information, including age, sex, and education, was obtained from each participant. The neurologic assessments, including disease duration, Unified Parkinson’s Disease Rating Scale (UPDRS), and Hoehn-Yahr stage, were recorded from all PD patients in practically defined OFF-medication condition (>12 hr after last dopaminergic medication). Additionally, motor symptoms were reevaluated in an ON-medication condition, defined as 1 hr following antiparkinsonian treatment (one tablet of immediate-release benserazide/levodopa 50/200 mg) immediately after initial clinical assessment and MRI scanning.

### MRI Data Acquisition and Processing

All participants were scanned on a 3.0 T MRI scanner (GE Health, Discovery 750) equipped with an eight-channel head coil. During MRI scanning, the head was stabilized using resting foam pads, and earplugs were provided to reduce the noise. Structural T1-weighted images were acquired using a fast spoiled gradient recalled sequence: repetition time (TR) = 7.336 ms; echo time (TE) = 3.036 ms; inversion time = 450 ms; flip angle (FA) = 11°; field of view (FOV) = 260 × 260 mm^2^; matrix = 256 × 256; slice thickness = 1.2 mm; 196 continuous sagittal slices. Rs-fMRI images were acquired using a gradient recalled echo–echo planar imaging sequence: TR= 2,000 ms; TE = 30 ms; FA = 77°; FOV = 240 × 240 mm^2^; matrix = 64 × 64; slice thickness = 4 mm; slice gap = 0 mm; 38 interleaved axial slices. After completing an initial rs-fMRI scanning session in the OFF-medication condition, PD patients were advised to take one tablet of benserazide/levodopa and were re-scanned one hour afterward.

The rs-fMRI data processing was performed using fMRIPrep v1.5.9 (https://fmriprep.org/en/1.5.9/; Esteban et al., 2019) with the default processing steps. To summarize: Each T1-weighted image was corrected for intensity nonuniformity and skull-stripped. Brain surfaces were reconstructed using *recon-all* from FreeSurfer software. Spatial normalization to the ICBM 152 Nonlinear Asymmetrical template version 2009c was performed through nonlinear registration, using brain-extracted versions of both the T1-weighted images and template. Brain tissue segmentation of cerebrospinal fluid, white matter, and gray matter was performed on the brain-extracted T1-weighted images. Functional data were corrected for slice-timing, motion, and field distortion. This was followed by coregistration to the corresponding T1-weighted images using boundary-based registration with 9 *df*. All processed rs-fMRI data were denoised by fMRIDenoise (https://github.com/compneuro-ncu/fmridenoise) with the *24HMP8PhysSpikeReg* pipeline, including temporal band-pass filtering (0.008–0.08 Hz), detrending, and confound regression. The confound regression employed 24 head motion parameters (three translations, three rotations, their temporal derivatives, and their quadratic term), eight physiological noise parameters (mean signals from white matter signal and cerebrospinal fluid, their temporal derivatives, and quadratic terms), and spike regressors based on framewise displacement (FD) and DVARS thresholds. After this, all functional data were resampled to 3-mm isotropic and smoothed with a 5-mm full width at half maximum (FWHM) Gaussian kernel, masked by gray matter.

### Network Construction

The functional network was constructed as in a previous study (Guan et al., 2019), where nodes represented brain regions and edges represented interregional functional connectivity between every pair of nodes. The anatomical automatic labeling (AAL) atlas with 90 regions of interest was used to generate network nodes. The mean time course of each node was extracted, and interregional resting-state functional connectivity was calculated based on the Pearson correlation between the time courses of each pair of nodes. To implement graph analyses relevant to the functional network, negative correlations were omitted, and networks were thresholded at a connection sparsity that showed the best discrimination ability of PD patients and normal controls (see the next section).

### Defining the Connection Sparsity for Network Analyses

Since the human brain network shows the characteristic of sparsity, each functional network was thresholded by applying a set of sparsity from 0.1 to 0.5 with an interval of 0.02. We used network density to refer sparsity, which is defined as the ratio of the number of connections existing in the network to the maximum possible number of connections (Liao, Vasilakos, & He, 2017). To implement the graph analyses in a specific sparse network, we employed support vector machine (SVM) to identify the specific sparsity for functional networks that was mostly discriminative and favorable for PD classification. We used the functional connectivity features of normal controls and PD patients in OFF-medication status to minimize the potential drug effects on the PD classification. To remove the redundant features, the functional connectivity features selected by a two-sample *t* test with *p* < 0.05 were applied in the SVM model using LIBSVM (https://www.csie.ntu.edu.tw/~cjlin/libsvm/) with default setting. Specifically, the hyperparameter C of SVM was set to 1, and radial basis function (RBF) kernel was used. The raw functional connectivity features were scaled individually to range [−1, +1]. A leave-one-out cross-validation (LOOCV) scheme was used to evaluate the performance of the SVM classifier. For each sparsity across the range of 0.1 to 0.5 with an interval of 0.02, the same SVM procedure was performed. The sparsity with the highest accuracy for PD classification was used to perform the network analyses. Statistical significance of the highest classification accuracy was determined by a permutation test, which involved repeating the classification procedure 1,000 times with a different random permutation of the training group labels (F. Li et al., 2014). Moreover, we also validated the results within the connection sparsity range of 0.1–0.5 with an interval of 0.1. The results are shown in the Supporting Information (Table S9 and Figures S1–S5).

### Network Analyses

The final functional network was thresholded by applying the sparsity with the best discriminative ability for PD classification. All network analyses were performed using Brain Connectivity Toolbox (BCT, https://sites.google.com/site/bctnet/).

#### Rich-club organization.

A brain network is thought to have a rich-club organization if nodes with a high degree are more densely and strongly interconnected than what would be expected by chance (van den Heuvel & Sporns, 2011). The presence of a rich-club organization was examined by calculating the weighted rich-club coefficient using BCT (Rubinov & Sporns, 2010). The weighted rich-club coefficient φ^w^(*k*) across a range of degree *k* of the individual brain network was computed; a detailed description is given in the Supporting Information. After the calculation of the weighted rich-club coefficient, the φ^w^(*k*) was normalized by comparing it with the mean weighted rich-club coefficient of 1,000 random networks. By definition, $\varphi_{\mathrm{norm}}^{w}$(*k*) > 1 for a range of *k* was indicative of a rich-club organization within a network.

#### Rich-club nodes and subnetwork analyses.

The rich-club node definition was based on normal controls. To define the rich-club nodes, a group-averaged network within the normal control group was computed as follows: First, from the set of individual group matrices, only connections that were present in at least 60% of the population of the group were selected for averages, while all other connections were set to 0. Then, the group-averaged matrix was computed by averaging only across the nonzero values of the individual subject matrices (R. Li et al., 2016). Based on the functional group-averaged network of normal controls, the rich-club regions were defined as the top 15% (*n* = 13) of brain regions with the highest degree (R. Li et al., 2016; Yan et al., 2018).

Identification of the rich-club regions allowed for the categorization of the whole-brain nodes of the connectome into three types: (a) rich-club nodes, the top 13 brain regions with the highest degree; (b) feeder nodes, showing the connections with rich-club nodes; and (c) peripheral nodes, the remaining nodes except rich-club nodes and feeder nodes. Based on these three types of nodes, we categorized three types of subnetworks: (a) rich-club subnetwork, a subgraph with rich-club nodes and the edges linking members of the rich-club nodes; (b) feeder subnetwork, a subgraph with feeder nodes and the edges linking members of the feeder nodes; and (c) peripheral subnetwork, a subgraph with peripheral nodes and the edges linking members of the peripheral nodes. These three types of nodes and the derived subnetworks based on normal controls were applied into the PD group in both OFF- and ON-medication status.

The further subnetwork analyses focused on these three types of nodes and subnetworks. First, we evaluated the nodal properties for three types of nodes, which were defined by the sum of degree centrality of all nodes belonging to a specific node category. Then, we assessed the intra-subnetwork properties for each type of subnetwork, including (a) network-based statistic (NBS, https://www.nitrc.org/projects/nbs/) analysis to identify the subnetwork difference between groups; (b) computation of the subnetwork connection strength, which was defined by the sum of all the weights of the connections within each type of subnetwork, respectively; and (c) calculation of the global efficiency for each type of subnetwork. Finally, we further analyzed the inter-subnetwork interactions, including (a) interactions between the rich-club subnetwork and feeder subnetwork, which were defined by the sum of all the weights of the connections linking the rich-club nodes and feeder nodes; and (b) interactions between the feeder subnetwork and peripheral subnetwork, which were defined by the sum of all the weights of the connections linking the feeder nodes and peripheral nodes.

#### Diverse-club nodes and subnetwork analyses.

In addition to the rich-club organization, there is another set of nodes that have edges diversely distributed across the network communities, forming a diverse club in human brain networks (Bertolero et al., 2017). The participation coefficient is an indicator of the diversity of each node’s connections across the network’s communities, where nodes with a high participation coefficient exhibit diverse connectivity and form the diverse club. Similar to the rich-club node definition, the diverse-club regions were generated based on normal control groups. We first detected the community structure based on the group-averaged network. Then, based on the community structure, we calculated the participation coefficient of each node. Similarly, we considered nodes with a high participation coefficient (top 15%) to be the diverse-club regions. The diverse-club nodes and the interconnections linking the members of diverse-club nodes comprised the diverse-club subnetwork, which was applied to PD groups. We calculated the connection strength and global efficiency of this diverse-club subnetwork.

### Statistical Analyses

Statistical analyses of demographic and clinical data were performed using SPSS 19.0 statistical software. The one-sample Kolmogorov-Smirnov test was used to check the data normality. Differences in the age, sex distribution, and education between groups were compared with the unpaired *t* tests, the Mann-Whitney U tests, and Pearson chi-squared test as appropriate. Wilcoxon signed-rank test was used to compare the difference of UPDRS motor scores between the OFF-medication condition and ON-medication condition. Statistical significance was set at *p* < 0.05.

To check whether a rich-club organization was present in the groups, a one-sample *t* test was performed at each level of *k* to examine whether the normalized rich-club coefficient $\varphi_{\mathrm{norm}}^{w}$(*k*) statistically exceeded 1 in each group separately. False discovery rate (FDR) correction was applied to correct for multiple comparison across all examined levels of *k*. To determine the significance levels of altered connectivity networks in NBS analysis, a general linear model controlling for age and sex as covariates at each edge independently was employed to test for group differences in subnetwork connectivity. A threshold (*p* = 0.05) was used to form a set of suprathreshold edges (connections) among which any connected components and their size (number of edges) could be determined. The statistical significance of the size of each observed component was assessed with respect to an empirical null distribution of maximal component sizes obtained under the null hypothesis of random group membership (5,000 permutations). Significant components in each subnetwork were determined at *p* < 0.05.

Group differences either in rich-club coefficient or other network properties between PD patients and normal controls were assessed using the permutation test (with 10,000 permutations) with age and sex as covariates. A paired *t* test or Wilcoxon signed-rank test was used appropriately to compare the network differences between patients in OFF-medication and ON-medication. Tests were two-tailed with a significance level of *p* < 0.05, and FDR correction (with *q* < 0.05) was applied to correct for multiple comparisons. Finally, the relationships between subnetwork properties or the clinical scores were examined via partial correlation analyses, taking age and sex into account. Specifically, the relationships between subnetwork properties and motor symptoms in OFF- and ON-medication status, as well as the relationships between subnetwork changes and motor symptom improvement, were examined. Statistical significance was set at *p* < 0.00185 (Bonferroni corrected, 3 subnetworks × 3 properties × 3 kinds of relationships, 27 correlations in total, *p* < 0.05/27 = 0.00185). Regarding the relationships between network properties, statistical significance was set at *p* < 0.0125 (Bonferroni corrected, 4 correlations in total: correlations between peripheral node degree and rich-club/feeder node degree in OFF-medication status, and correlations between peripheral node degree change rate and rich-club/feeder node degree improvement rate after levodopa administration).

In order to assess whether the results could be explained by motion, we also conducted permutation tests with age, sex, and mean FD as covariates. Further, correlation analyses between motion parameters and network metrics in both the normal control group and patients in either OFF or ON status were performed. Results are shown in the Supporting Information (Tables S4 and S5). The comparison results with mean FD as covariates between groups were similar to the main results; in addition, there were no correlations between motion parameters and network metrics in both the control group and PD patients whether in OFF or ON status (FDR corrected), indicating that the network alterations were not explained by motion.

### Atlas-Based Validation on Hierarchical Topology

A functional brain network is constructed by defining the synchronization of rs-fMRI signals between predefined brain regions, which could be potentially affected by the different brain parcellations (atlas) employed in the computations. To minimize the potential influence of brain atlas selection, we recruited a newly constructed brain atlas (200 parcellations) that was derived from rs-fMRI data and that was in agreement with certain architectonic and visuotopic boundaries (https://github.com/ThomasYeoLab/CBIG/tree/master/stable_projects/brain_parcellation/Schaefer2018_LocalGlobal; Schaefer et al., 2018) to validate our results. Based on this atlas, we replicated the same procedures to define different levels of the brain hierarchical system and explored the neural substrate of immediate levodopa effect from the view of brain hierarchical configuration.

## RESULTS

### Demographic and Clinical Characteristics

Demographic and clinical features of PD patients and normal controls are shown in Table 1. Age, sex distribution, or education was not significantly different between PD patients and normal controls. After levodopa administration, motor symptoms were significantly relieved in PD patients (*p* < 0.001).

**Table 1.** Demographic and clinical information

	Normal controls	Parkinson’s disease patients	*p* value
Age, mean (*SD*)	60.6 (7.0)	60.9 (8.8)	0.805^a^
Sex (M/F)	42/47	35/26	0.220^b^
Education, mean (*SD*)	8.5 (3.3)	8.1 (4.6)	0.504^c^
Disease duration, mean (*SD*)	–	4.7 (3.6)	–
UPDRS-III (OFF/ON), mean (*SD*)	–	23.6 (15.0)/15.4 (12.5)	<0.001^d^
Hoehn-Yahr stage, median (range)	–	2.5 (1–5)	–

^a^ Unpaired *t* tests.

^b^ Pearson chi-squared test.

^c^ Mann-Whitney U tests.

^d^ Wilcoxon signed-rank test.

### Connection Sparsity for Network Analyses

The final functional networks were thresholded at a sparsity of 0.2, in which the discriminative ability for PD classification reached the highest accuracy of 88.7% (Table 2) and was highly significant at *p* = 0.001. Thus, all of the following network analyses were performed based on the network sparsity of 0.2.

**Table 2.** Accuracy for PD classification across different sparsities

Sparsity	Accuracy (%)	Sparsity	Accuracy (%)	Sparsity	Accuracy (%)
0.10	88	0.24	86	0.38	80
0.12	88	0.26	85.3	0.40	79.3
0.14	86.7	0.28	85.3	0.42	78
0.16	86	0.30	85.3	0.44	78.7
0.18	85.3	0.32	84.7	0.46	76
0.20	**88.7**	0.34	83.3	0.48	78
0.22	86.7	0.36	82	0.50	78

### Rich-Club Organization

**Figure 1.**
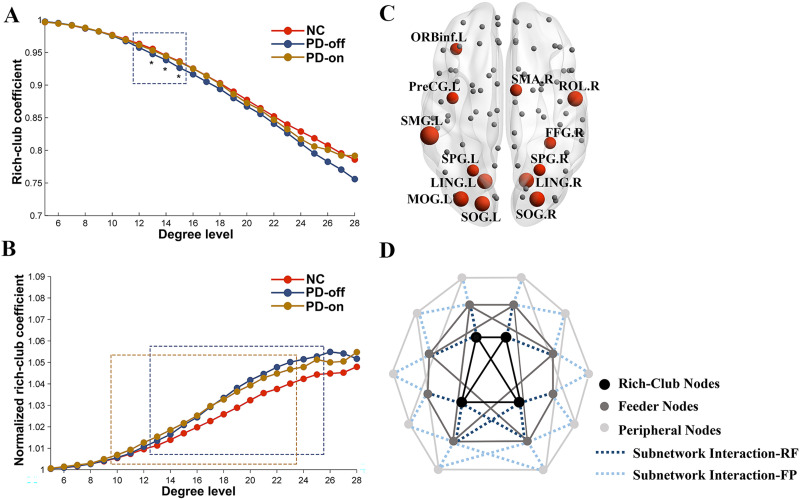
Rich-club organization of functional connectome. Group-averaged rich-club curve of weighted rich-club coefficient (A) and normalized weighted rich-club coefficient (B) for NC (red), PD-off (blue), and PD-on (yellow). The dashed box indicates the differences between NC and PD patients (blue for PD-off, yellow for PD-on) after the FDR correction. * indicates the difference between PD-off and PD-on with *p* < 0.05. (C) Red nodes represent the functional rich-club regions. This figure is based on the functional group-averaged network in controls. The size of the red nodes indicates the degree centrality. (D) A simplified example of the three types of nodes: rich-club nodes (black nodes), feeder nodes (gray nodes), and peripheral nodes (light gray nodes). Three types of nodes could form three classes of subnetworks. Dashed lines indicate the subnetwork interaction (dark blue represents the interactions between rich-club subnetwork and feeder subnetwork; light blue shows the interactions between feeder subnetwork and peripheral subnetwork). Abbreviations: NC, normal controls; PD-off, PD patients in OFF-medication condition; PD-on, PD patients in ON-medication condition; ORBinf.L, left orbital part of inferior frontal gyrus; PreCG.L, left precentral gyrus; SMG.L, left supramarginal gyrus; SPG.L, left superior parietal gyrus; LING.L, left lingual gyrus; MOG.L, left middle occipital gyrus; SOG.L, left superior occipital gyrus; SMA.R, right supplementary motor area; ROL.R, right Rolandic operculum; FFG.R, right fusiform; SPG.R, right superior parietal gyrus; LING.R, right lingual gyrus; SOG.R, right superior occipital gyrus; subnetwork interaction-RF, interactions between rich-club subnetwork and feeder subnetwork; subnetwork interaction-FP, interactions between feeder subnetwork and peripheral subnetwork.

Figure 1 depicts the averaged rich-club coefficients and normalized rich-club coefficients for both groups. In the whole-brain network, the rich-club coefficient was significantly lower in PD patients than in normal controls in the range *k* = 12–15 (FDR corrected); after the levodopa administration, the rich-club coefficient showed no difference between PD patients and normal controls. Compared with the OFF-medication condition, patients in the ON-medication condition showed an increased rich-club coefficient in the range *k* = 13–15 (*p* < 0.05, uncorrected; Figure 1A). The normalized rich-club coefficient increases as a function of degree (*k*) higher than 1 in both normal controls and PD patients in the OFF- and ON-medication condition (Figure 1B), indicating a rich-club organization of the functional network in both groups. Comparisons of the normalized rich-club coefficient showed that PD patients in either OFF-medication condition or ON-medication condition exhibited a higher normalized rich-club coefficient than normal controls in the range *k* = 13–25 for OFF-medication condition and *k* = 10–23 for ON-medication condition, FDR corrected. No difference was observed for the normalized rich-club coefficient in PD patients between OFF-medication and ON-medication conditions.

#### Rich-club nodes.

The rich-club nodes, selected on the basis of the group-averaged network, ranking top 15% highest degree, included the following regions: left precentral gyrus, left orbital part of inferior frontal gyrus, right Rolandic operculum, right supplementary motor area, bilateral lingual gyrus, bilateral superior occipital gyrus, left middle occipital gyrus, right fusiform, bilateral superior parietal gyrus, and left supramarginal gyrus (Figure 1C, red nodes).

#### Intra-subnetwork analyses.

**Figure 2.**
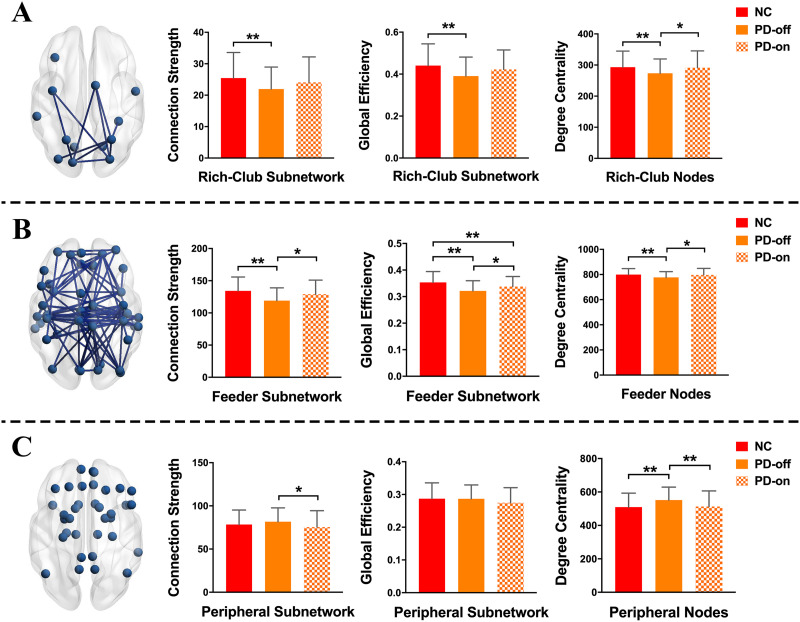
Comparisons of network properties for the rich-club subnetwork (A), feeder subnetwork (B), and peripheral subnetwork (C). The blue nodes represent three types of nodes identified in this study. The edges concatenating nodes in (A) and (B) indicate a significant component detected by network-based statistic (NBS) analysis in the rich-club subnetwork and feeder subnetwork, respectively. ** indicates the differences corrected by FDR correction; * indicates the uncorrected differences with *p* < 0.05.

Results of the intra-subnetwork analyses are shown in Figure 2 and Supporting Information Table S1. First, in the comparison between normal controls and PD patients in OFF-medication condition, NBS analysis revealed a component showing significantly lower functional connectivity in PD patients in the rich-club subnetwork (*p* = 0.008) and feeder subnetwork (*p* = 0.0002), respectively (Figure 2, column 1). Analyses of the intra-subnetwork connection strength showed that in the OFF-medication condition, PD patients exhibited decreased functional connectivity strength in the rich-club subnetwork (*p* = 0.0019) and feeder subnetwork (*p* < 0.001) compared with normal controls. There was no difference in connection strength in the peripheral subnetwork between PD patients in the OFF-medication condition and normal controls (*p* = 0.1205). After levodopa administration, there was no difference in connection strength between PD patients and normal controls in three types of subnetworks (*p* = 0.1326, 0.0526, and 0.1390 for rich-club subnetwork, feeder subnetwork, and peripheral subnetwork, respectively). Compared with the OFF-medication condition, PD patients in the ON-medication condition showed increased connection strength in the feeder subnetwork (*p* = 0.009) and decreased connection strength in the peripheral subnetwork (*p* = 0.0383) (Figure 2, column 2, and Supporting Information Table S1). These results indicated that levodopa administration could improve the disrupted functional connection strength in the rich-club subnetwork and feeder subnetwork.

Regarding the global efficiency of the subnetwork, compared with normal controls, PD patients in the OFF-medication condition showed decreased global efficiency in the rich-club subnetwork and feeder subnetwork (both *p* < 0.001); PD patients in the ON-medication condition showed decreased global efficiency in the feeder subnetwork (*p* = 0.0061). Compared with the OFF-medication condition, PD patients in the ON-medication condition exhibited increased global efficiency in the feeder subnetwork (*p* = 0.0203). No difference for global efficiency in the peripheral subnetwork was observed between normal controls and PD patients in either the OFF-medication condition or the ON-medication condition (*p* = 0.48 and 0.0531, respectively; Figure 2, column 3, and Supporting Information Table S1). Similarly, levodopa administration relieved the damaged efficiency in the rich-club subnetwork and feeder subnetwork.

#### Nodal property analyses.

For the analyses of nodal property, compared with normal controls, we found that PD patients in the OFF-medication condition showed decreased nodal degree centrality in rich-club nodes and feeder nodes (*p* = 0.0075 and 0.0029, respectively), and increased degree centrality in peripheral nodes (*p* < 0.001); no difference was observed between patients in the ON-medication condition and normal controls in three types of nodes (*p* = 0.4188, 0.3745, and 0.3840 for rich-club nodes, feeder nodes, and peripheral nodes, respectively). Compared with the OFF-medication condition, patients in the ON-medication condition showed increased nodal degree centrality in rich-club nodes and feeder nodes (*p* = 0.0209 and 0.0257, respectively), which suggested a corrected effect of levodopa on rich-club nodes and feeder nodes; intriguingly, the degree centrality in peripheral nodes was decreased (*p* = 0.0130), which suggested a potentially compensatory effect of peripheral nodes on PD pathology (Figure 2, column 4, and Supporting Information Table S1).

#### Inter-subnetwork interaction.

**Figure 3.**
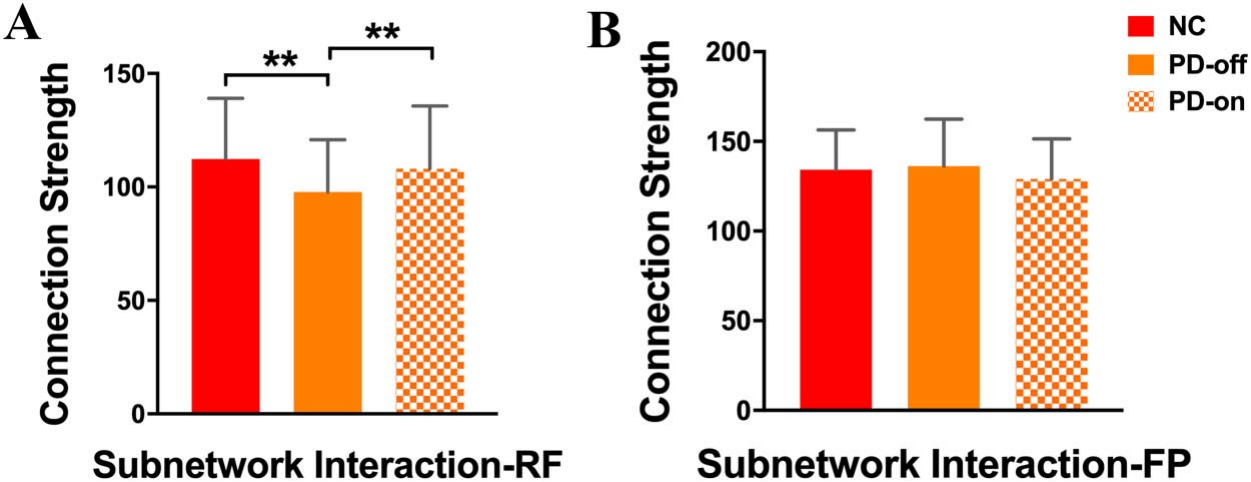
Comparisons of subnetwork interactions between the rich-club subnetwork and feeder subnetwork (A), and between the feeder subnetwork and peripheral subnetwork (B). ** indicates the differences corrected by FDR correction.

We analyzed the interactions between subnetworks. We found that compared with normal controls, the interactions between the rich-club subnetwork and feeder subnetwork was decreased in PD patients in the OFF-medication condition (*p* < 0.001), while PD patients in the ON-medication condition and normal controls showed no difference in these interactions (*p* = 0.1687); directly compared with PD patients in the OFF-medication condition, patients in the ON-medication condition exhibited increased interactions between the rich-club and feeder subnetwork (*p* = 0.0202; Figure 3A and Supporting Information Table S2). For the interactions between the feeder subnetwork and peripheral subnetwork, there was no difference between normal controls and patients in either the OFF-medication condition or ON-medication condition (*p* = 0.3018 and 0.0893, respectively; Figure 3B and Supporting Information Table S2).

#### Relationships among network properties and clinical scores.

**Figure 4.**
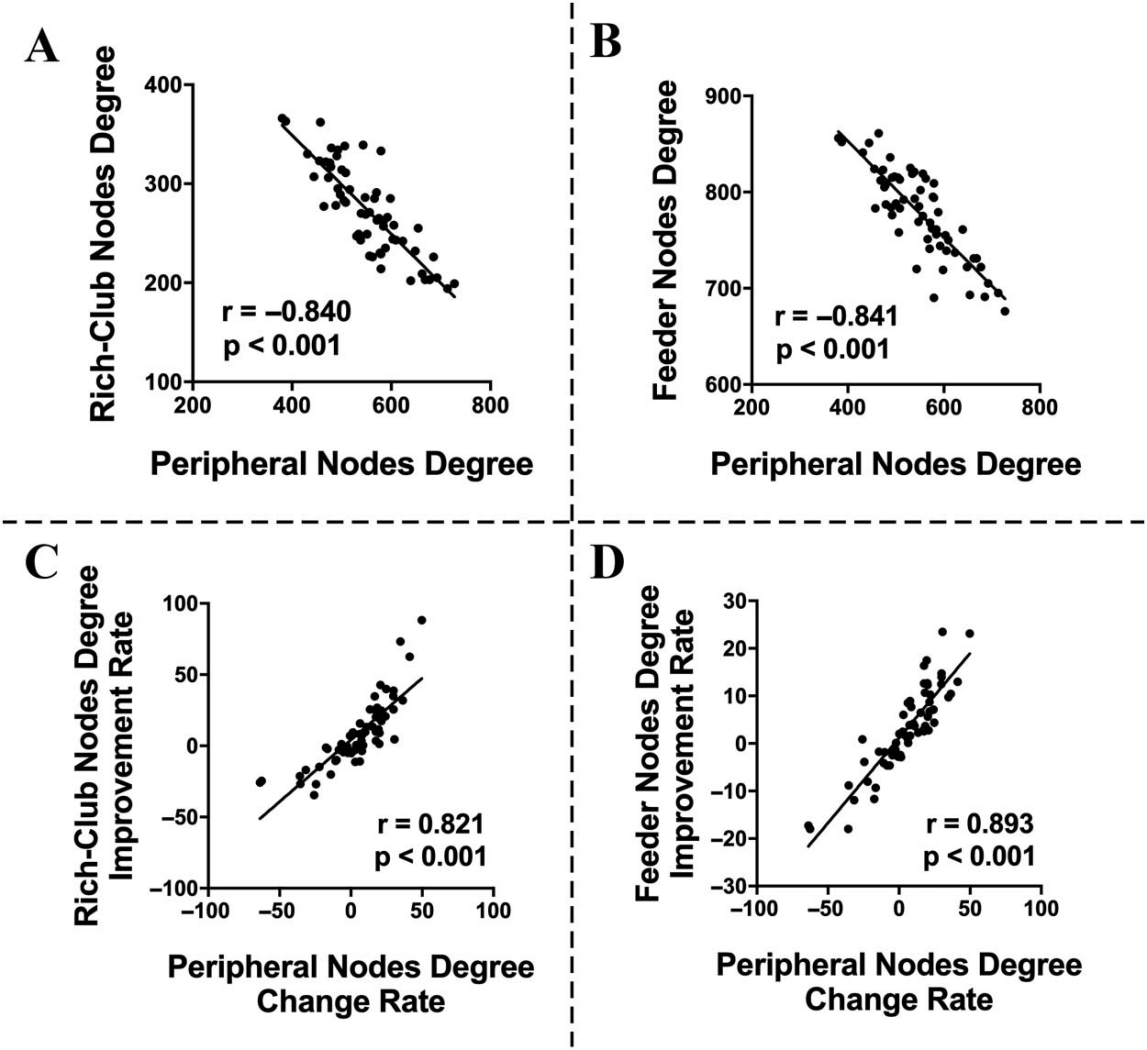
Correlations between network properties. Negative correlations between peripheral node degree and rich-club node degree (A) or feeder nodes degree (B) in OFF-medication condition. Positive correlations between the change rate of the peripheral node degree and the improvement rate of rich-club node degree (C) and feeder node degree (D).

We did not find any relationships between subnetwork properties and motor symptom scores in PD patients in either the OFF-medication condition or the ON-medication condition, and there was no correlation between network changes and motor symptom improvements (Supporting Information Table S8). Interestingly, we found that in the OFF-medication condition, the degree centrality of peripheral nodes was negatively correlated with degree centrality of both rich-club nodes and feeder nodes (*r* = −0.840, *p* < 0.001, and *r* = −0.841, *p* < 0.001, respectively; Figures 4A and 4B). After levodopa administration, the change rate of degree in peripheral nodes, computed as (degree_OFF_ − degree_ON_)/degree_OFF_, was positively correlated with the improvement rate of the degree in both rich-club nodes and feeder nodes, computed as (degree_ON_ − degree_OFF_)/degree_OFF_ (*r* = 0.821, *p* < 0.001, and *r* = 0.893, *p* < 0.001, respectively; Figures 4C and 4D). These relationships between nodal degree centrality suggested that the peripheral nodes may serve as a positive role to compensate the disrupted function of core nodes (including rich-club nodes and feeder nodes).

### Diverse-Club Analyses

**Figure 5.**
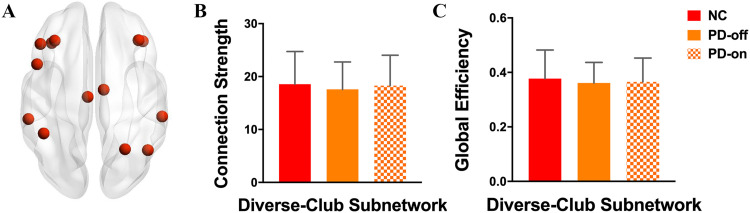
Comparisons of diverse-club properties among groups. (A) Red nodes represent diverse-club regions in the brain. (B) Connection strength and (C) global efficiency difference within the diverse-club subnetwork were compared among groups.

The diverse-club nodes, selected by the top 15% highest participation coefficients based on the group-averaged network, included the following regions: bilateral middle frontal gyrus, left opercular part of inferior frontal gyrus, left triangular part of inferior frontal gyrus, bilateral orbital part of inferior frontal gyrus, bilateral middle cingulum, right superior parietal gyrus, left inferior gyrus, bilateral supramarginal gyrus, and right angular (Figure 5A, red nodes).

Analyzing the connection strength and the global efficiency within the diverse-club subnetwork, we found that there was no difference between normal controls and PD patients in either the OFF-medication condition or the ON-medication condition (Figures 5B and 5C, and Supporting Information Table S3), indicating a relatively reserved function of the diverse club in PD patients.

### Robustness of Normalization Effect of Levodopa on Hierarchical Brain Organization in PD

We used the same network analysis procedures by employing another atlas to validate the main findings. First, both normal controls and PD patients in the OFF- and ON- medication status showed a rich-club organization in their functional network, as the normalized rich-club coefficient higher than 1 across a range of degree (Supporting Information Figure S6). Second, we analyzed hierarchical brain organization along the rich-club core structure as in previous procedures. We observed impaired function within the rich-club and feeder subnetwork in PD patients with OFF-medication status, represented by decreased connection strength and global efficiency, as well as decreased nodal degree within rich-club and feeder subnetworks. For the peripheral subnetwork, PD patients in OFF-medication status showed increased connection strength and nodal degree. After levodopa administration, these abnormal network properties tend to reach a relatively normal state (Supporting Information Table S11). In summary, by recruiting a new brain atlas, we found similar hierarchical brain topography and levodopa normalizing effects as the previous findings, verifying that these results were independent of the specific brain parcellations.

## DISCUSSION

In this study, we applied a graph theory–based approach to analyze the topological organization of the functional connectome in PD patients and the impact of dopaminergic therapy on its functional reorganization. The main findings were as follows: (a) PD patients in the OFF-medication condition showed impaired global network property for rich-club organization, while the diverse club preserved function, indicating a differentiated brain topological organization in PD patients. (b) Decreased nodal degree centrality in rich-club nodes and feeder nodes were observed in PD patients in the OFF-medication condition, while the peripheral nodal degree showed an increase, suggesting the distinct functional patterns in different types of nodes. And (c) levodopa could exert a normalizing effect on abnormal network architecture of the rich-club system.

### Differentiated Brain Topological Organization in PD Patients

Rich-club organization is a property common to complex networks and is considered to be a basis for efficient global information transfer and complex neurological function in the brain (van den Heuvel et al., 2012; van den Heuvel & Sporns, 2011). In the present study, the functional brain networks of both normal controls and PD patients showed a rich-club organization, which meant that normal controls as well as PD patients both had a subset of highly connected nodes that were more interconnected than what would be expected by chance (van den Heuvel & Sporns, 2011). This finding was consistent with previous research reporting the existence of rich-club organization in PD patients (C. Li et al., 2017). Notwithstanding the presence of a rich-club organization, PD patients showed a significant reduction in rich-club interconnectivity compared with normal controls, which suggested that it was difficult for patients to maintain or repair this core subnetwork composed of rich-club nodes. Previous studies had showed that the high-degree nodes had higher blood flow, higher glucose metabolic rate, and longer connection distance than other nodes (Collin, Sporns, Mandl, & van den Heuvel, 2014; Tomasi, Wang, & Volkow, 2013). Their high topological centrality and high biological cost could make these nodes particularly vulnerable to pathogenic factors (Crossley et al., 2014). This phenomenon was strengthened by studies in different clinical populations, for example, in multiple sclerosis (Stellmann et al., 2017), schizophrenia (van den Heuvel et al., 2013), and epilepsy (R. Li et al., 2016). Our findings of disrupted interconnectivity among rich-club regions were in line with the previous evidence and supported the proposal that the high-degree regions are generally more susceptible to the pathology of PD. Additionally, the existence of a rich-club organization has been proposed to underlie important network properties, such as global efficiency (Xu, Zhang, & Small, 2010). The topologically central role of the rich club may indicate that pathological attack on pivotal regions will have an impact on the network’s global efficiency of information processing (Albert, Jeong, & Barabasi, 2000). Therefore, the reduced global efficiency of the rich-club subnetwork suggests that the detected abnormalities may be partly due to impaired rich-club organization.

As opposed to the high connectivity that high-degree nodes exhibited, nodes with a high participation coefficient exhibited diverse connectivity. These nodes are also highly interconnected and form a diverse club (Bertolero et al., 2017). In the present study, in contrast to the rich club, the connection strength and global efficiency of the diverse club showed no disruption in PD, indicating a preserved function of the diverse club. The diverse club is another topological configuration of the human brain network showing distinct roles in network communication that differ from the rich club. Specifically, the function of the rich club may predominately be to maintain stability in the entire network via slow processing, potentially using its high degree to integrate information at slower timescales, while the diverse club may act on shorter timescales (Bertolero et al., 2017; Gollo, Zalesky, Hutchison, van den Heuvel, & Breakspear, 2015). These different functions of the rich club and the diverse club may underlie the observed phenomenon—impaired rich club and preserved diverse club—indicating that the topological dysfunction in PD has an intrinsic, distinct pattern.

### Distinct Functional Pattern Attributes of Different Levels of Nodes

Identification of rich-club nodes allows us to class the whole-brain nodes into different levels, including rich-club nodes, feeder nodes, and peripheral nodes. Subsequently, the whole-brain network could be subdivided into three subnetworks based on node classification. In the present study, we observed reduced connection strength and global efficiency within the rich-club and feeder subnetwork, whereas the properties within the peripheral subnetwork were unaffected, which suggested that PD is characterized by selective disruption in central node-related brain configuration. As mentioned before, the rich-club subnetwork and to a lesser extent the feeder network are biologically costly, making it harder to maintain or further develop, and therefore they are more likely to be affected in the pathological condition (Collin et al., 2014; Crossley et al., 2014). On the other hand, the peripheral network might have a lower biological cost and therefore is less vulnerable and less affected in the same pathological burden (Verhelst et al., 2018). Therefore, our results underline the importance of subdividing the brain into subnetworks and uncovering differential effects of PD pathology on the hierarchical brain subnetworks’ properties.

Further, analyses of the nodal property reinforced the proposal of distinct functional patterns for different levels of nodes. A differential pattern of nodal degree centrality was observed in three levels of nodes. In particular, we found that PD patients in OFF-medication status showed a decrease of nodal degree in rich-club nodes and feeder nodes together with an increase of nodal degree in peripheral nodes. These results suggested a likely divergence of nodal function in hierarchical network architecture. Since the rich-club nodes and to a lesser extent the feeder nodes are more likely to exhibit pathology (Crossley et al., 2014), the reduction of nodal degree in rich-club and feeder nodes may represent the direct pathologic influence. The increased degree centrality in peripheral nodes might reflect an attempt to restore or compensate reduced rich-club and feeder node degree. The human brain is an integrative network; brain nodes are working together to maintain the overall function of whole brain. Given the vulnerability of topology of central nodes under the pathologic condition, other nodes with a lower biological cost (e.g., peripheral nodes) may up-regulate their function to balance the overall communication within the whole brain (Crossley et al., 2014; Verhelst et al., 2018). A similar mechanism was reported in patients with traumatic brain injury (Verhelst et al., 2018); the authors found that the increased strength was confined to the local subnetwork and may compensate the reduced rich-club connectivity. Interestingly, the inverse associations between reduced degree centrality in rich-club nodes as well as feeder nodes and increased degree centrality in peripheral nodes were observed in the present study. This relationship further theoretically supported the notion of peripheral nodes’ compensatory role. Taken together, analyses of nodal properties as well as subnetwork properties suggested a distinct functional pattern for different levels of nodes: disrupted function in topological central-related nodes accompanied by a compensatory effect in topological peripheral nodes.

Since different network sparsities may influence the network analyses, we repeated the same network analyses over a range of network sparsities (0.1:0.1:0.5). As a result, over the range of sparsity thresholds, normalized rich-club coefficients of normal controls and PD patients in either OFF- or ON-medication status were more than 1, indicating the existence of the rich-club organization in both groups. Moreover, the findings of impaired function of rich-club and feeder subnetworks in PD OFF-medication status consistently existed over the range of sparsity thresholds. The changes in peripheral subnetworks in PD patients with OFF-medication status were increased overall (e.g., increased peripheral node degree) along the range of sparsity thresholds, indicating a compensatory role of the peripheral subnetwork, while the specific metric (e.g., global efficiency) of the peripheral subnetwork showed a decrement when the network was denser (corresponding to a higher sparsity, e.g., sparsity = 0.4/0.5). The human brain network shows an ability to balance cost and efficiency; a denser network often indicates a higher wiring cost, which could result in a decrement of network efficiency (Liao et al., 2017). In our study, we showed that the core hierarchical structures of the network were consistently impaired along a range of sparsity in PD patients, which was consistent with our main findings and may be a result of the PD pathology regardless of the network density. Similarly, the peripheral subnetwork with lower biological cost exhibited an overall compensatory effect under the PD pathology, while the slight variation of efficiency at a higher density reflected the flexibility to dynamically balance the network cost (density) and the network efficiency, which further indicated a lower vulnerability of the peripheral subnetwork under the same pathological burden.

### Levodopa Modulates Abnormal Network Architecture

To further investigate the effect of dopaminergic medication on the abnormal brain network, we compared the normal controls and PD patients in ON-medication status. The improvement of decreased network properties in the rich-club subnetwork and feeder subnetwork were observed after levodopa administration. Furthermore, the direct comparisons of network properties between OFF- and ON-medication conditions yielded a significant increment of network properties mainly in the feeder subnetwork. These results implied that levodopa administration could improve the disrupted brain topology.

Previous studies investigated the effect of dopaminergic therapy on brain function (Esposito et al., 2013; Gao, Zhang, Chan, & Wu, 2017; Zhong et al., 2019), and the results showed that levodopa had a significant impact on restoring impaired functional connectivity of the sensorimotor network, default mode network, and basal ganglia motor circuit. The findings in this study were consistent with the previous results and indicated the restorative effects of levodopa on brain function. In addition, we found that levodopa could reduce the increased degree centrality of peripheral nodes in the OFF-medication condition, making the brain’s organization reach a relatively normal state. Consistent with our study, Berman et al. (2016) found that levodopa can reduce the local efficiency of specific subnetworks that show significant increments in the PD OFF-medication state, suggesting a normalizing effect of levodopa on brain topology. Thus, these findings indicated that levodopa could modulate abnormal brain architecture; it could not only improve impaired brain function but also normalize the abnormally increased brain topological properties. Furthermore, a positive correlation between the change rate of peripheral node degree and the improvement rate of rich-club (and feeder) node degree was observed in this study; this finding reinforced the speculation of the compensatory role of peripheral nodes as we discussed above, and also suggested that levodopa could impart a flexible modulation effect on different parts of the brain’s organization, making the whole brain reach a normal state.

### Limitations

Several limitations of this study should be acknowledged. One major limitation was that PD patients only took levodopa without any placebo, and normal controls were scanned only one time. Although we ensured that all participants were in a relatively stable state, the time effects may potentially influence the results. Both drug, placebo, and time effects should be considered in future studies to optimize the experimental design. Second, there may be a long duration response for dopaminergic drugs since we only withdraw antiparkinsonian drugs for at least 12 hr; even though this withdrawal time has been widely used to reflect PD OFF-medication status (Albanese et al., 2001; Zach et al., 2020). Third, this study was a cross-sectional study with a moderate sample size; further prospective and longitudinal studies with a larger sample size are warranted to validate these findings and, importantly, to explore the longitudinal alterations of the different types of nodes along the disease progression, which is expected to give an in-depth understanding of the topological organization in PD patients. Finally, PD patients in this study were under multiple antiparkinsonian treatment, which may have a potential influence on the investigation of levodopa effect; future studies including drug-naïve PD patients could purify the effect of levodopa and contribute to exploring the levodopa-induced brain alteration.

### Conclusion

This study revealed differentiated brain organization in PD patients: The function of the rich-club organization was disrupted, while the function of the diverse club was preserved. Furthermore, a functional divergence existed in the PD hierarchical brain system, characterized by disrupted function in topological central nodes along with the compensatory effect in topological peripheral nodes. Finally, dopaminergic therapy could modulate the brain architecture to make it reach a normal state.

## ACKNOWLEDGMENTS

The authors thank all the normal volunteers and Parkinson’s disease patients recruited in this project. The authors appreciate clinical assistance from other neurologists in the Department of Neurology at the Second Affiliated Hospital of Zhejiang University School of Medicine.

## SUPPORTING INFORMATION

Supporting information for this article is available at https://doi.org/10.1162/netn_a_00232.

## AUTHOR CONTRIBUTIONS

Tao Guo: Conceptualization; Data curation; Formal analysis; Writing – original draft; Writing – review & editing. Min Xuan: Conceptualization; Data curation; Formal analysis; Writing – original draft; Writing – review & editing. Cheng Zhou: Data curation. Jingjing Wu: Data curation. Ting Gao: Data curation. Xueqin Bai: Data curation. Xiaocao Liu: Data curation. Luyan Gu: Data curation. Ruiqi Liu: Software. Zhe Song: Writing – review & editing. Quanquan Gu: Funding acquisition; Writing – review & editing. Peiyu Huang: Funding acquisition; Writing – review & editing. Jiali Pu: Investigation. Baorong Zhang: Investigation. Xiaojun Xu: Writing – review & editing. Xiaojun Guan: Data curation; Funding acquisition. Minming Zhang: Conceptualization; Funding acquisition; Supervision; Writing – review & editing.

## FUNDING INFORMATION

Minming Zhang, 13th Five-Year Plan for National Key Research and Development Program of China, Award ID: 2016YFC1306600. Minming Zhang, National Natural Science Foundation of China (https://dx.doi.org/10.13039/501100001809), Award ID: 81971577. Xiaojun Guan, National Natural Science Foundation of China (https://dx.doi.org/10.13039/501100001809), Award ID: 82001767. Xiaojun Guan, China Postdoctoral Science Foundation (https://dx.doi.org/10.13039/501100002858), Award ID: 2021T140599 and 2019M662082. Xiaojun Xu, National Natural Science Foundation of China (https://dx.doi.org/10.13039/501100001809), Award ID: 82171888. Xiaojun Guan, Natural Science Foundation of Zhejiang Province (https://dx.doi.org/10.13039/501100004731), Award ID: LQ21H180008. Min Xuan, Natural Science Foundation of Zhejiang Province (https://dx.doi.org/10.13039/501100004731), Award ID: LQ20H180012.

## TECHNICAL TERMS

Brain hubs: Some brain regions play a central role in the overall network organization, as indexed by a high degree or high participation in multiple communities across the network.
Rich club: When the hubs of a network tend to be more densely connected among themselves than nodes of a lower degree, the network present a rich-club organization.
Diverse club: Nodes with a high participation coefficient are connected densely, forming the diverse club.
Rich-club nodes: Here, defined as the top 13 brain regions with the highest degree.
Feeder nodes: Showing the connections with rich-club nodes.
Peripheral nodes: The remaining nodes except rich-club nodes and feeder nodes in the whole brain.
