# Supplementary material for: Normalization effect of levodopa on hierarchical brain function in Parkinson’s disease
Source: Netw Neurosci. 2022 Jun 1;6(2):552–69. doi: 10.1162/netn_a_00232 (PMC9208001; doi:10.1162/netn_a_00232)
Supplement: Supplementary file 1 [file netn-06-552-s001.pdf]

Guo, T., Xuan, M., Zhou, C., Wu, J., Gao, T., Bai, X., Liu, X., Gu, L., Liu, R., Song, Z., Gu, Q., Huang, P., Pu, J., Zhang, B., Xu, X., Guan, X. & Zhang, M. (2022). Supporting information for "Normalization effect of levodopa on hierarchical brain function in Parkinson's disease." *Network Neuroscience*. Advance publication. https://doi.org/10.1162/netn_a_00232

Supplementary Materials

Normalization effect of levodopa on hierarchical brain function in Parkinson's disease

2 Materials and Methods

2.5 Network analyses

The final functional network was thresholded by applying the sparsity with the best discriminative ability for PD classification. All network analyses were performed using Brain Connectivity Toolbox (BCT, https://sites.google.com/site/bctnet/).

2.5.1 Rich-club organization

A brain network is thought to have a rich-club organization if nodes with a high degree are more densely and stronger interconnected than would be expected by chance (1). The presence of a rich-club organization was examined by calculating the weighted rich-club coefficient using BCT (2). The weighted rich-club coefficient $\varphi^{w}(k)$ across a range of degree *k* of the individual brain network was computed. For this, firstly, a subgraph was created with only the nodes having a degree larger than *k*. Then, the number of edges *n* connecting the nodes in the subgraph was counted and their weights were summed resulting in the strength of the subgraph. Next, the edges

of the total network were ranked according to their weight and the weights of the *n* strongest edges were summed. The weighted rich-club coefficient was calculated by dividing the strength of the subgraph by the strength of the n strongest edges of the total network. After the calculation of the weighted rich-club coefficient, the $\varphi^{w}(k)$ was normalized by comparing it to the mean weighted rich-club coefficient of 1000 random networks. By definition, $\varphi^{w}_{norm}(k) > 1$ for a range of *k* is indicative of a rich club organization within a network.

Table S1 Rich-club derived intra-subnetwork comparisons between groups.

	Normal controls (mean ± SD)	PD patients (mean ± SD)		Comparisons (difference/p value) [a]		
		Off-medication	On-medication	NC vs. PD-off	NC vs. PD-on	PD-off vs. PD-on
**Rich-club subnetwork**						
Connection strength	25.44 ± 8.15	21.93 ± 7.01	24.09 ± 8.09	3.710/0.0019	1.4814/0.1326	-1.7428/0.0865
Global efficiency	0.44 ± 0.10	0.39 ± 0.09	0.42 ± 0.09	0.0520/5.9994e^{-4}	0.0200/0.1126	-1.9286/0.0585
Nodes degree	293.3 ± 51.4	273.5 ± 45.9	291.7 ± 53.8	20.4962/0.0075	1.7095/0.4188	-2.3728/0.0209
**Feeder subnetwork**						
Connection strength	134.3 ± 21.4	119.2 ± 19.76	129.1 ± 21.88	15.4124/9.999e^{-5}	5.4150/0.0526	-2.6979/0.0090
Global efficiency	0.35 ± 0.04	0.32 ± 0.04	0.34 ± 0.04	0.0330/9.999e^{-5}	0.0162/0.0061	-2.3837/0.0203
Nodes degree	799.3 ± 48.2	777 ± 46.1	797.3 ± 51.1	22.5576/0.0029	2.5103/0.3745	-2.2880/0.0257
**Peripheral subnetwork**						
Connection strength	78.45 ± 16.78	81.7 ± 15.99	75.34 ± 19.02	-3.2358/0.1205	3.1257/0.1390	2.1183/0.0383
Global efficiency	0.29 ± 0.05	0.29 ± 0.04	0.27 ± 0.05	9.9418e^{-4}/0.48	0.0127/0.0531	1.5495/0.1265
Nodes degree	509.4 ± 83.7	551.5 ± 77.3	513.0 ± 93.0	-43.0538/7.9992e^{-4}	-4.2198/0.3840	2.5588/0.0130

[a] For PD-off vs. PD-on, difference was presented by stats value.

Table S2 Comparisons of rich-club derived subnetwork interaction between groups.

Subnetwork Interaction	Normal controls (mean ± SD)	PD patients (mean ± SD)		Comparisons (difference/p value) [a]		
		Off-medication	On-medication	NC vs. PD-off	NC vs. PD-on	PD-off vs. PD-on
Interaction-RF	112.3 ± 26.7	97.8 ± 23.0	108.0 ± 27.7	14.8714/3.9996e^{-4}	4.3070/0.1687	-2.3860/0.0202
Interaction-FP	134.2 ± 22.2	136.3 ± 26.1	129.1 ± 22.4	-2.0799/0.3018	5.0679/0.0893	1.8968/0.0627

[a] For PD-off vs. PD-on, difference was presented by stats value.

Abbreviation: Interaction-RF, interactions between rich-club subnetwork and feeder subnetwork; Interaction-FP, interactions between feeder subnetwork and peripheral subnetwork.

Table S3 Comparisons of diverse-club subnetwork between groups.

Subnetwork<br>Interaction	Normal controls<br>(mean ± SD)	PD patients (mean ± SD)		Comparisons (difference/p value) [a]		
		Off-medication	On-medication	NC vs. PD-off	NC vs. PD-on	PD-off vs. PD-on
Connection strength	18.56 ± 6.17	17.58 ± 5.19	18.25 ± 5.76	1.2415/0.0953	0.5847/0.2766	-0.8125/0.4197
Global efficiency	0.38 ± 0.10	0.36 ± 0.08	0.37 ± 0.09	0.02/0.0919	0.0163/0.1586	-0.2929/0.7706

[a] For PD-off vs. PD-on, difference was presented by stats value.

Table S4 Rich-club derived intra-subnetwork comparisons between normal controls and PD patients with age, sex, and mean FD as covariates.

	Rich-club subnetwork (p value)			Feeder subnetwork (p value)			Peripheral subnetwork (p value)		
	Connection strength	Global efficiency	Nodes degree	Connection strength	Global efficiency	Nodes degree	Connection strength	Global efficiency	Nodes degree
NC vs. PD-off	0.0013	0.0015	0.0071	$9.999e^{-5}$	$9.999e^{-5}$	0.0023	0.1006	0.4691	$6.9993e^{-4}$
NC vs. PD-on	0.1001	0.0927	0.3427	0.0716	0.0081	0.3136	0.2290	0.0749	0.2948

Table S5 Correlations between network metrics and motion parameters.

	Translation_x			Translation_y			Translation_z		
	NC	PD-off	PD-on	NC	PD-off	PD-on	NC	PD-off	PD-on
**Rich-club subnetwork**									
Global efficiency	0.013/0.904	-0.076/0.559	-0.004/0.976	0.184/0.084	0.011/0.936	-0.138/0.289	-0.180/0.091	-0.096/0.461	0.036/0.783
Connection strength	0.004/0.970	-0.103/0.431	-0.015/0.908	0.167/0.119	-0.007/0.957	-0.084/0.519	-0.142/0.185	-0.063/0.629	0.010/0.939
Nodes degree	0.022/0.836	-0.139/0.286	-0.040/0.758	0.170/0.111	-0.074/0.568	-0.013/0.923	-0.097/0.365	-0.134/0.304	0.064/0.624
**Feeder subnetwork**									
Global efficiency	0.056/0.600	-0.001/0.994	0.063/0.632	0.066/0.541	-0.020/0.879	-0.158/0.225	-0.216/0.042	0.173/0.183	0.090/0.492
Connection strength	-0.007/0.946	-0.064/0.622	0.071/0.588	0.122/0.253	-0.136/0.295	-0.025/0.847	-0.159/0.136	0.027/0.834	0.138/0.288
Nodes degree	-0.052/0.626	-0.017/0.900	0.065/0.620	0.119/0.268	-0.029/0.823	0.036/0.784	-0.200/0.060	0.122/0.349	0.139/0.284
**Peripheral subnetwork**									
Global efficiency	0.033/0.758	0.200/0.122	0.000/0.999	-0.212/0.046	0.063/0.630	-0.086/0.508	0.063/0.558	0.132/0.309	-0.132/0.310
Connection strength	0.060/0.576	0.007/0.955	-0.046/0.726	-0.156/0.145	0.027/0.837	-0.038/0.770	0.156/0.145	-0.016/0.901	-0.154/0.237
Nodes degree	0.008/0.939	0.092/0.479	-0.024/0.852	-0.169/0.112	0.019/0.887	-0.024/0.852	0.157/0.141	0.007/0.959	-0.114/0.384

Data was present as original r/p.

Table S5 (continue)

	Rotation_x			Rotation_y			Rotation_z		
	NC	PD-off	PD-on	NC	PD-off	PD-on	NC	PD-off	PD-on
**Rich-club subnetwork**									
Global efficiency	0.033/0.760	-0.100/0.443	-0.082/0.528	0.051/0.636	-0.144/0.269	-0.026/0.843	0.015/0.886	0.031/0.812	0.134/0.304
Connection strength	-0.015/0.887	-0.212/0.100	-0.067/0.608	-0.026/0.808	-0.143/0.273	0.003/0.982	-0.021/0.842	0.059/0.651	0.123/0.345
Nodes degree	-0.054/0.613	-0.147/0.259	0.033/0.798	-0.054/0.614	-0.032/0.807	0.043/0.741	0.016/0.880	0.028/0.831	0.144/0.268
**Feeder subnetwork**									
Global efficiency	-0.097/0.367	0.066/0.612	-0.205/0.113	0.077/0.474	-0.089/0.495	0.019/0.886	0.020/0.849	-0.014/0.917	0.169/0.193
Connection strength	-0.117/0.273	-0.041/0.751	-0.002/0.987	0.124/0.248	-0.075/0.567	-0.073/0.578	-0.083/0.437	0.062/0.634	0.185/0.154
Nodes degree	-0.116/0.279	0.093/0.477	0.155/0.234	0.121/0.258	-0.248/0.054	-0.093/0.478	-0.162/0.130	0.105/0.419	0.220/0.089
**Peripheral subnetwork**									
Global efficiency	0.087/0.419	0.085/0.516	-0.109/0.402	-0.061/0.573	0.146/0.262	-0.032/0.806	0.059/0.580	-0.047/0.718	-0.121/0.351
Connection strength	0.097/0.366	0.037/0.780	-0.155/0.233	0.006/0.958	0.251/0.051	0.045/0.731	0.070/0.515	-0.182/0.160	-0.186/0.150
Nodes degree	0.123/0.252	0.032/0.807	-0.118/0.364	-0.029/0.786	0.191/0.140	0.026/0.843	0.083/0.437	-0.074/0.572	-0.195/0.132

Data was present as original r/p.

Table S5 (continue)

	mean FD		
	NC	PD-off	PD-on
**Rich-club subnetwork**			
Global efficiency	-0.115/0.283	0.097/0.459	-0.030/0.819
Connection strength	-0.155/0.147	0.103/0.430	-0.143/0.270
Nodes degree	-0.211/0.048	0.107/0.412	-0.139/0.287
**Feeder subnetwork**			
Global efficiency	-0.024/0.822	0.252/0.050	-0.033/0.803
Connection strength	-0.099/0.355	0.245/0.058	-0.027/0.835
Nodes degree	-0.169/0.114	0.102/0.433	-0.110/0.400
**Peripheral subnetwork**			
Global efficiency	0.176/0.098	-0.160/0.219	0.145/0.264
Connection strength	0.201/0.058	0.005/0.967	0.166/0.202
Nodes degree	0.210/0.048	-0.114/0.380	0.121/0.352

Data was present as original r/p.

Table S6 Comparisons of motion parameters between controls and patients.

	Comparisons (p value)		
	Controls vs. PD-off	Controls vs. PD-on	PD-off vs. PD-on
Mean FD	0.405	0.005	0.006
Translation_x	0.895	0.965	0.525
Translation_y	0.883	<0.001	0.001
Translation_z	0.064	0.443	0.571
Rotation_x	0.907	0.407	0.293
Rotation_y	0.784	0.802	0.674
Rotation_z	0.645	0.656	0.395
Percentage of spikes	0.725	0.101	0.327

Table S7 Rich-club derived intra-subnetwork comparisons between groups (comparisons of normalized metrics).

	NC vs. PD-off		NC vs. PD-on		PD-off vs. PD-on [a]	
	difference	P value	difference	P value	difference	P value
**Rich-club subnetwork**						
Connection strength	0.4687	0.0017 *	0.1871	0.1329	-1.7428	0.0865
Global efficiency	0.5267	$8.9991e^{-4}$ *	0.2022	0.1113	-1.9286	0.0585
Nodes degree	0.4010	0.0063*	0.0334	0.4249	-2.3728	0.0209*
**Feeder subnetwork**						
Connection strength	0.7033	$9.999e^{-5}$ *	0.2471	0.0556	-2.6979	0.0090*
Global efficiency	0.7987	$9.999e^{-5}$ *	0.3914	0.0028*	-2.3837	0.0203*
Nodes degree	0.4583	0.0023*	0.0510	0.3720	-2.2880	0.0257*
**Peripheral subnetwork**						
Connection strength	-0.1867	0.1204	0.1804	0.1417	2.1183	0.0383*
Global efficiency	0.0086	0.4827	0.2761	0.0502	1.5495	0.1265
Nodes degree	-0.4985	$7.9992e^{-4}$ *	-0.0489	0.3792	2.5588	0.0130*

[a] For PD-off vs. PD-on, difference was presented by stats value. * indicates $p < 0.05$.

Table S8 Relationship between network properties and motor symptom.

		Rich-club subnetwork			Feeder subnetwork			Peripheral subnetwork		
		Connection strength	Global efficiency	Nodes degree	Connection strength	Global efficiency	Nodes degree	Connection strength	Global efficiency	Nodes degree
Relationships between network properties and motor symptom in OFF status										
UPDRS-III scores	r	-0.162	-0.140	-0.053	-0.128	-0.178	-0.064	0.141	-0.002	0.069
	p	0.220	0.289	0.692	0.336	0.177	0.630	0.287	0.988	0.601
Relationships between network properties and motor symptom in ON status										
UPDRS-III scores	r	-0.102	-0.06	-0.104	-0.138	-0.09	-0.181	0.177	0.117	0.160
	p	0.441	0.653	0.434	0.299	0.497	0.169	0.180	0.378	0.225
Relationships between network changes and motor symptom improvement										
Motor improvement	r	-0.148	-0.160	-0.221	-0.094	-0.033	-0.052	-0.066	-0.235	-0.144
	p	0.264	0.227	0.093	0.480	0.802	0.695	0.617	0.073	0.278

Notes: motor improvement was calculated by $OFF_{UPDRS\text{-}III}$- $ON_{UPDRS\text{-}III}$; network changes of rich-club subnetwork and feeder subnetwork were calculated by $ON_{metrics}$-$OFF_{metrics}$; network changes of peripheral subnetwork were calculated by $OFF_{metrics}$-$ON_{metrics}$.

Table S9 Rich-club derived intra-subnetwork comparisons between groups across a range of sparsity thresholds.

		NC vs. PD-off		NC vs. PD-on		PD-off vs. PD-on [a]	
		difference	P value	difference	P value	difference	P value
**Sparsity = 0.1**	**Rich-club subnetwork**						
	Connection strength	1.5654	0.0563	0.3033	0.3831	-1.3250	0.1902
	Global efficiency	0.0439	0.0072*	0.0196	0.1459	-1.3629	0.1780
	Nodes degree	6.3149	0.1229	-2.5068	0.3303	-1.7813	0.0799
	**Feeder subnetwork**						
	Connection strength	9.1409	$9.999e^{-5}$*	2.2037	0.1259	-3.1721	0.0024*
	Global efficiency	0.0270	$2.9997e^{-4}$*	0.0131	0.0255*	-1.8099	0.0753
	Nodes degree	15.5526	$3.9996e^{-4}$*	0.8914	0.4314	-2.5863	0.0121*
	**Peripheral subnetwork**						
	Connection strength	-3.2369	0.0455*	1.7092	0.1923	2.1696	0.0340*
	Global efficiency	-0.0144	0.0639	0.0119	0.1202	2.6055	0.0116*
	Nodes degree	-21.8675	0.0042*	1.6154	0.4266	2.4682	0.0164

[a] For PD-off vs. PD-on, difference was presented by stats value. * indicates $p < 0.05$.

Table S9 (continue)

		NC vs. PD-off		NC vs. PD-on		PD-off vs. PD-on [a]	
		difference	P value	difference	P value	difference	P value
**Sparsity = 0.3**	**Rich-club subnetwork**						
	Connection strength	4.4737	$4.9995e^{-4}$*	1.6716	0.1250	-2.0987	0.0401*
	Global efficiency	0.0502	$8.9991e^{-4}$*	0.0186	0.1190	-2.2328	0.0293*
	Nodes degree	28.7401	0.0034*	4.4542	0.3412	-2.5716	0.0126*
	**Feeder subnetwork**						
	Connection strength	21.4063	$9.999e^{-5}$*	9.8344	0.0209*	-2.2803	0.0262*
	Global efficiency	0.0371	$9.999e^{-5}$*	0.0201	0.0039*	-2.1460	0.0359*
	Nodes degree	27.0718	0.0092*	6.1162	0.2849	-1.9722	0.0532
	**Peripheral subnetwork**						
	Connection strength	-1.9075	0.2912	4.7573	0.0914	1.9725	0.0532
	Global efficiency	0.0101	0.0528	0.0164	0.0069*	1.1855	0.2405
	Nodes degree	-55.8119	0.0018*	-10.5704	0.2811	2.4635	0.0166*

[a] For PD-off vs. PD-on, difference was presented by stats value. * indicates $p < 0.05$.

Table S9 (continue)

		NC vs. PD-off		NC vs. PD-on		PD-off vs. PD-on [a]	
		difference	P value	difference	P value	difference	P value
**Sparsity = 0.4**	**Rich-club subnetwork**						
	Connection strength	4.9842	3.9996e-4*	2.1683	0.0699	-2.0744	0.0423*
	Global efficiency	0.0562	2.9997e-4*	0.0246	0.0573	-2.1698	0.0340*
	Nodes degree	31.8665	0.0024*	4.6467	0.3413	-2.6840	0.0094*
	**Feeder subnetwork**						
	Connection strength	26.7531	1.9998e-4*	13.9199	0.0128*	-2.0330	0.0465*
	Global efficiency	0.0416	9.9999e-5*	0.0224	0.0051*	-2.1521	0.0354*
	Nodes degree	30.1440	0.0140*	9.4428	0.2337	-1.7025	0.0938
	**Peripheral subnetwork**						
	Connection strength	0.2825	0.4689	7.1141	0.0397*	1.8325	0.0718
	Global efficiency	0.0148	0.0160*	0.0177	0.0052*	0.6228	0.5358
	Nodes degree	-62.0105	0.0021*	-14.0896	0.2579	2.3324	0.0231*

[a] For PD-off vs. PD-on, difference was presented by stats value. * indicates $p < 0.05$.

Table S9 (continue)

		NC vs. PD-off		NC vs. PD-on		PD-off vs. PD-on [a]	
		difference	P value	difference	P value	difference	P value
**Sparsity = 0.5**	**Rich-club subnetwork**						
	Connection strength	5.1061	$3.9996e^{-4}$*	2.1840	0.0695	-2.1043	0.0395*
	Global efficiency	0.0566	$9.999e^{-5}$*	0.0260	0.0485*	-2.0785	0.0419*
	Nodes degree	31.009	0.0058*	4.4395	0.3553	-2.5607	0.0130*
	**Feeder subnetwork**						
	Connection strength	31.6625	$9.999e^{-5}$*	17.8372	0.0089*	-1.8639	0.0672
	Global efficiency	0.0416	$9.999e^{-5}$*	0.0235	0.0057*	-1.9348	0.0577
	Nodes degree	33.8978	0.0140*	13.1596	0.1843	-1.6273	0.1089
	**Peripheral subnetwork**						
	Connection strength	2.8303	0.2688	8.8357	0.0255*	1.5403	0.1287
	Global efficiency	0.0169	0.0094*	0.0193	0.004*	0.5008	0.6184
	Nodes degree	-64.9068	0.0047*	-17.5991	0.2260	2.2501	0.0281*

[a] For PD-off vs. PD-on, difference was presented by stats value. * indicates $p < 0.05$.

Table S10 Comparisons of tDOF-loss and spike regressors between groups.

	Normal controls (mean ± SD)	PD patients (mean ± SD)		Comparisons (p value)		
		Off-medication	On-medication	NC vs. PD-off	NC vs. PD-on	PD-off vs. PD-on
tDOF-loss[a]	35.63 ± 7.21	36.30 ± 10.79	35.51 ± 9.51	0.725	0.093	0.342
Number of spike regressors	3.63 ± 7.2	4.30 ± 10.8	3.51 ± 9.51	0.725	0.093	0.342
Percentage of spike regressors	1.77 ± 3.52	2.10 ± 5.26	1.71 ± 4.64	0.725	0.101	0.327

[a] tDOF-loss: the loss of temporal degrees of freedom, which was calculated as the number of nuisance regressors input to the general linear model used to model noise in the BOLD data.

Table S11 Rich-club derived intra-subnetwork comparisons between groups using Schaefer2018_200 parcel atlas.

	Normal controls (mean ± SD)	PD patients (mean ± SD)		Comparisons (difference/p value) [a]		
		Off-medication	On-medication	NC vs. PD-off	NC vs. PD-on	PD-off vs. PD-on
**Rich-club subnetwork**						
Connection strength	220.33 ± 53.16	205.16 ± 52.71	221.05 ± 54.67	16.3636/0.0318*	0.9997/0.4510	-1.7777/0.0805
Global efficiency	0.53 ± 0.08	0.49 ± 0.08	0.52 ± 0.09	0.0368/0.0028*	0.0082/0.2749	-2.0273/0.0471*
Nodes degree	2446 ± 300	2362 ± 324	2496 ± 340	87.6276/0.0463*	-42.1537/0.2053	-2.4536/0.0171*
**Feeder subnetwork**						
Connection strength	887.34 ± 148.70	792.41 ± 126.61	862.49 ± 160.11	97.7821/9.999e^{-5}*	25.9398/0.1489	-2.9674/0.0043*
Global efficiency	0.37 ± 0.04	0.34 ± 0.04	0.36 ± 0.05	0.0317/9.999e^{-5}*	0.0111/0.0682	-2.7547/0.0078*
Nodes degree	6028 ± 256	5911 ± 292	6047 ± 286	120.9757/0.0051*	-19.3255/0.3296	-2.8826/0.0055*
**Peripheral subnetwork**						
Connection strength	443.66 ± 72.23	475.14 ± 114.80	431.25 ± 89.89	-31.0296/0.0227*	12.5295/0.1747	2.7830/0.0072*
Global efficiency	0.34 ± 0.03	0.34 ± 0.03	0.33 ± 0.03	0.0043/0.1831	0.0112/0.0057*	1.6339/0.1075
Nodes degree	3466 ± 492	3668 ± 534	3396 ± 566	-208.6033/0.0080*	61.4792/0.2437	3.1260/0.0027*

[a] For PD-off vs. PD-on, difference was presented by stats value. * indicates $p < 0.05$.

Table S12 Subnetwork division of AAL atlas.

Labels	Regions	Subnetworks	Labels	Regions	Subnetworks	Labels	Regions	Subnetworks
1	Precentral_L	Rich-club	11	Frontal_Inf_Oper_L	Feeder	21	Olfactory_L	Peripheral
2	Precentral_R	Feeder	12	Frontal_Inf_Oper_R	Peripheral	22	Olfactory_R	Peripheral
3	Frontal_Sup_L	Peripheral	13	Frontal_Inf_Tri_L	Feeder	23	Frontal_Sup_Medial_L	Feeder
4	Frontal_Sup_R	Peripheral	14	Frontal_Inf_Tri_R	Peripheral	24	Frontal_Sup_Medial_R	Peripheral
5	Frontal_Sup_Orb_L	Feeder	15	Frontal_Inf_Orb_L	Rich-club	25	Frontal_Med_Orb_L	Peripheral
6	Frontal_Sup_Orb_R	Feeder	16	Frontal_Inf_Orb_R	Feeder	26	Frontal_Med_Orb_R	Peripheral
7	Frontal_Mid_L	Peripheral	17	Rolandic_Oper_L	Feeder	27	Rectus_L	Feeder
8	Frontal_Mid_R	Peripheral	18	Rolandic_Oper_R	Rich-club	28	Rectus_R	Feeder
9	Frontal_Mid_Orb_L	Feeder	19	Supp_Motor_Area_L	Feeder	29	Insula_L	Feeder
10	Frontal_Mid_Orb_R	Feeder	20	Supp_Motor_Area_R	Rich-club	30	Insula_R	Feeder

Table S12 (continue)

Labels	Regions	Subnetworks	Labels	Regions	Subnetworks	Labels	Regions	Subnetworks
31	Cingulum_Ant_L	Peripheral	41	Amygdala_L	Peripheral	51	Occipital_Mid_L	Rich-club
32	Cingulum_Ant_R	Peripheral	42	Amygdala_R	Peripheral	52	Occipital_Mid_R	Feeder
33	Cingulum_Mid_L	Feeder	43	Calcarine_L	Feeder	53	Occipital_Inf_L	Feeder
34	Cingulum_Mid_R	Feeder	44	Calcarine_R	Feeder	54	Occipital_Inf_R	Feeder
35	Cingulum_Post_L	Peripheral	45	Cuneus_L	Feeder	55	Fusiform_L	Feeder
36	Cingulum_Post_R	Peripheral	46	Cuneus_R	Feeder	56	Fusiform_R	Rich-club
37	Hippocampus_L	Peripheral	47	Lingual_L	Rich-club	57	Postcentral_L	Feeder
38	Hippocampus_R	Peripheral	48	Lingual_R	Rich-club	58	Postcentral_R	Feeder
39	ParaHippocampal_L	Peripheral	49	Occipital_Sup_L	Rich-club	59	Parietal_Sup_L	Rich-club
40	ParaHippocampal_R	Feeder	50	Occipital_Sup_R	Rich-club	60	Parietal_Sup_R	Rich-club

Table S12 (continue)

Labels	Regions	Subnetworks	Labels	Regions	Subnetworks	Labels	Regions	Subnetworks
61	Parietal_Inf_L	Feeder	71	Caudate_L	Peripheral	81	Temporal_Sup_L	Feeder
62	Parietal_Inf_R	Feeder	72	Caudate_R	Peripheral	82	Temporal_Sup_R	Feeder
63	SupraMarginal_L	Rich-club	73	Putamen_L	Peripheral	83	Temporal_Pole_Sup_L	Feeder
64	SupraMarginal_R	Feeder	74	Putamen_R	Peripheral	84	Temporal_Pole_Sup_R	Peripheral
65	Angular_L	Peripheral	75	Pallidum_L	Peripheral	85	Temporal_Mid_L	Feeder
66	Angular_R	Peripheral	76	Pallidum_R	Peripheral	86	Temporal_Mid_R	Peripheral
67	Precuneus_L	Peripheral	77	Thalamus_L	Peripheral	87	Temporal_Pole_Mid_L	Peripheral
68	Precuneus_R	Peripheral	78	Thalamus_R	Peripheral	88	Temporal_Pole_Mid_R	Peripheral
69	Paracentral_Lobule_L	Feeder	79	Heschl_L	Feeder	89	Temporal_Inf_L	Feeder
70	Paracentral_Lobule_R	Feeder	80	Heschl_R	Feeder	90	Temporal_Inf_R	Feeder

Table S13 Subnetwork division of Schaefer2018_200_7Networks parcel atlas.

Labels	Regions	Subnetworks	Labels	Regions	Subnetworks	Labels	Regions	Subnetworks
1	LH_Vis_1	Peripheral	11	LH_Vis_11	Feeder	21	LH_SomMot_7	Rich-Club
2	LH_Vis_2	Feeder	12	LH_Vis_12	Peripheral	22	LH_SomMot_8	Rich-Club
3	LH_Vis_3	Feeder	13	LH_Vis_13	Peripheral	23	LH_SomMot_9	Feeder
4	LH_Vis_4	Peripheral	14	LH_Vis_14	Feeder	24	LH_SomMot_10	Feeder
5	LH_Vis_5	Feeder	15	LH_SomMot_1	Feeder	25	LH_SomMot_11	Rich-Club
6	LH_Vis_6	Peripheral	16	LH_SomMot_2	Feeder	26	LH_SomMot_12	Feeder
7	LH_Vis_7	Peripheral	17	LH_SomMot_3	Feeder	27	LH_SomMot_13	Rich-Club
8	LH_Vis_8	Feeder	18	LH_SomMot_4	Rich-Club	28	LH_SomMot_14	Rich-Club
9	LH_Vis_9	Peripheral	19	LH_SomMot_5	Rich-Club	29	LH_SomMot_15	Feeder
10	LH_Vis_10	Peripheral	20	LH_SomMot_6	Feeder	30	LH_SomMot_16	Feeder

Table S13 (continue)

Labels	Regions	Subnetworks	Labels	Regions	Subnetworks	Labels	Regions	Subnetworks
31	LH_DorsAttn_Post_1	Feeder	41	LH_DorsAttn_FEF_1	Feeder	51	LH_SalVentAttn_PFCl_1	Peripheral
32	LH_DorsAttn_Post_2	Feeder	42	LH_DorsAttn_FEF_2	Feeder	52	LH_SalVentAttn_Med_1	Feeder
33	LH_DorsAttn_Post_3	Feeder	43	LH_DorsAttn_PrCv_1	Feeder	53	LH_SalVentAttn_Med_2	Feeder
34	LH_DorsAttn_Post_4	Rich-Club	44	LH_SalVentAttn_ParOper_1	Feeder	54	LH_SalVentAttn_Med_3	Rich-Club
35	LH_DorsAttn_Post_5	Rich-Club	45	LH_SalVentAttn_ParOper_2	Rich-Club	55	LH_Limbic_OFC_1	Peripheral
36	LH_DorsAttn_Post_6	Feeder	46	LH_SalVentAttn_ParOper_3	Rich-Club	56	LH_Limbic_OFC_2	Peripheral
37	LH_DorsAttn_Post_7	Feeder	47	LH_SalVentAttn_FrOperIns_1	Feeder	57	LH_Limbic_TempPole_1	Feeder
38	LH_DorsAttn_Post_8	Rich-Club	48	LH_SalVentAttn_FrOperIns_2	Feeder	58	LH_Limbic_TempPole_2	Feeder
39	LH_DorsAttn_Post_9	Feeder	49	LH_SalVentAttn_FrOperIns_3	Feeder	59	LH_Limbic_TempPole_3	Peripheral
40	LH_DorsAttn_Post_10	Rich-Club	50	LH_SalVentAttn_FrOperIns_4	Feeder	60	LH_Limbic_TempPole_4	Peripheral

Table S13 (continue)

Labels	Regions	Subnetworks	Labels	Regions	Subnetworks	Labels	Regions	Subnetworks
61	LH_Cont_Par_1	Feeder	71	LH_Cont_pCun_1	Peripheral	81	LH_Default_Par_3	Peripheral
62	LH_Cont_Par_2	Feeder	72	LH_Cont_Cing_1	Peripheral	82	LH_Default_Par_4	Peripheral
63	LH_Cont_Par_3	Rich-Club	73	LH_Cont_Cing_2	Peripheral	83	LH_Default_PFC_1	Peripheral
64	LH_Cont_Temp_1	Feeder	74	LH_Default_Temp_1	Peripheral	84	LH_Default_PFC_2	Peripheral
65	LH_Cont_OFC_1	Peripheral	75	LH_Default_Temp_2	Peripheral	85	LH_Default_PFC_3	Peripheral
66	LH_Cont_PFCl_1	Peripheral	76	LH_Default_Temp_3	Peripheral	86	LH_Default_PFC_4	Peripheral
67	LH_Cont_PFCl_2	Peripheral	77	LH_Default_Temp_4	Peripheral	87	LH_Default_PFC_5	Feeder
68	LH_Cont_PFCl_3	Feeder	78	LH_Default_Temp_5	Feeder	88	LH_Default_PFC_6	Peripheral
69	LH_Cont_PFCl_4	Feeder	79	LH_Default_Par_1	Peripheral	89	LH_Default_PFC_7	Peripheral
70	LH_Cont_PFCl_5	Feeder	80	LH_Default_Par_2	Feeder	90	LH_Default_PFC_8	Feeder

Table S13 (continue)

Labels	Regions	Subnetworks	Labels	Regions	Subnetworks	Labels	Regions	Subnetworks
91	LH_Default_PFC_9	Peripheral	101	RH_Vis_1	Feeder	111	RH_Vis_11	Feeder
92	LH_Default_PFC_10	Peripheral	102	RH_Vis_2	Feeder	112	RH_Vis_12	Feeder
93	LH_Default_PFC_11	Peripheral	103	RH_Vis_3	Feeder	113	RH_Vis_13	Feeder
94	LH_Default_PFC_12	Peripheral	104	RH_Vis_4	Peripheral	114	RH_Vis_14	Feeder
95	LH_Default_PFC_13	Feeder	105	RH_Vis_5	Feeder	115	RH_Vis_15	Feeder
96	LH_Default_pCunPCC_1	Peripheral	106	RH_Vis_6	Feeder	116	RH_SomMot_1	Feeder
97	LH_Default_pCunPCC_2	Peripheral	107	RH_Vis_7	Peripheral	117	RH_SomMot_2	Feeder
98	LH_Default_pCunPCC_3	Peripheral	108	RH_Vis_8	Feeder	118	RH_SomMot_3	Feeder
99	LH_Default_pCunPCC_4	Peripheral	109	RH_Vis_9	Peripheral	119	RH_SomMot_4	Rich-Club
100	LH_Default_PHC_1	Peripheral	110	RH_Vis_10	Peripheral	120	RH_SomMot_5	Rich-Club

Table S13 (continue)

Labels	Regions	Subnetworks	Labels	Regions	Subnetworks	Labels	Regions	Subnetworks
121	RH_SomMot_6	Feeder	131	RH_SomMot_16	Rich-Club	141	RH_DorsAttn_Post_7	Rich-Club
122	RH_SomMot_7	Feeder	132	RH_SomMot_17	Feeder	142	RH_DorsAttn_Post_8	Feeder
123	RH_SomMot_8	Feeder	133	RH_SomMot_18	Feeder	143	RH_DorsAttn_Post_9	Rich-Club
124	RH_SomMot_9	Rich-Club	134	RH_SomMot_19	Feeder	144	RH_DorsAttn_Post_10	Rich-Club
125	RH_SomMot_10	Feeder	135	RH_DorsAttn_Post_1	Rich-Club	145	RH_DorsAttn_FEF_1	Rich-Club
126	RH_SomMot_11	Rich-Club	136	RH_DorsAttn_Post_2	Feeder	146	RH_DorsAttn_FEF_2	Feeder
127	RH_SomMot_12	Feeder	137	RH_DorsAttn_Post_3	Rich-Club	147	RH_DorsAttn_PrCv_1	Rich-Club
128	RH_SomMot_13	Rich-Club	138	RH_DorsAttn_Post_4	Feeder	148	RH_SalVentAttn_TempOccPar_1	Feeder
129	RH_SomMot_14	Feeder	139	RH_DorsAttn_Post_5	Rich-Club	149	RH_SalVentAttn_TempOccPar_2	Feeder
130	RH_SomMot_15	Rich-Club	140	RH_DorsAttn_Post_6	Feeder	150	RH_SalVentAttn_TempOccPar_3	Rich-Club

Table S13 (continue)

Labels	Regions	Subnetworks	Labels	Regions	Subnetworks	Labels	Regions	Subnetworks
151	RH_SalVentAttn_PrC_1	Feeder	161	RH_Limbic_OFC_3	Peripheral	171	RH_Cont_PFCl_2	Feeder
152	RH_SalVentAttn_FrOperIns_1	Peripheral	162	RH_Limbic_TempPole_1	Peripheral	172	RH_Cont_PFCl_3	Feeder
153	RH_SalVentAttn_FrOperIns_2	Feeder	163	RH_Limbic_TempPole_2	Peripheral	173	RH_Cont_PFCl_4	Feeder
154	RH_SalVentAttn_FrOperIns_3	Feeder	164	RH_Limbic_TempPole_3	Feeder	174	RH_Cont_PFCl_5	Feeder
155	RH_SalVentAttn_FrOperIns_4	Feeder	165	RH_Cont_Par_1	Feeder	175	RH_Cont_PFCl_6	Peripheral
156	RH_SalVentAttn_Med_1	Rich-Club	166	RH_Cont_Par_2	Feeder	176	RH_Cont_PFCl_7	Feeder
157	RH_SalVentAttn_Med_2	Feeder	167	RH_Cont_Par_3	Feeder	177	RH_Cont_pCun_1	Feeder
158	RH_SalVentAttn_Med_3	Feeder	168	RH_Cont_Temp_1	Feeder	178	RH_Cont_Cing_1	Peripheral
159	RH_Limbic_OFC_1	Peripheral	169	RH_Cont_PFCv_1	Peripheral	179	RH_Cont_Cing_2	Feeder
160	RH_Limbic_OFC_2	Peripheral	170	RH_Cont_PFCl_1	Feeder	180	RH_Cont_PFCmp_1	Feeder

Table S13 (continue)

Labels	Regions	Subnetworks	Labels	Regions	Subnetworks
181	RH_Cont_PFCmp_2	Peripheral	191	RH_Default_PFCdPFCm_1	Peripheral
182	RH_Default_Par_1	Peripheral	192	RH_Default_PFCdPFCm_2	Peripheral
183	RH_Default_Par_2	Peripheral	193	RH_Default_PFCdPFCm_3	Peripheral
184	RH_Default_Par_3	Peripheral	194	RH_Default_PFCdPFCm_4	Peripheral
185	RH_Default_Temp_1	Peripheral	195	RH_Default_PFCdPFCm_5	Peripheral
186	RH_Default_Temp_2	Peripheral	196	RH_Default_PFCdPFCm_6	Peripheral
187	RH_Default_Temp_3	Peripheral	197	RH_Default_PFCdPFCm_7	Peripheral
188	RH_Default_Temp_4	Peripheral	198	RH_Default_pCunPCC_1	Peripheral
189	RH_Default_Temp_5	Peripheral	199	RH_Default_pCunPCC_2	Peripheral
190	RH_Default_PFCv_1	Feeder	200	RH_Default_pCunPCC_3	Peripheral

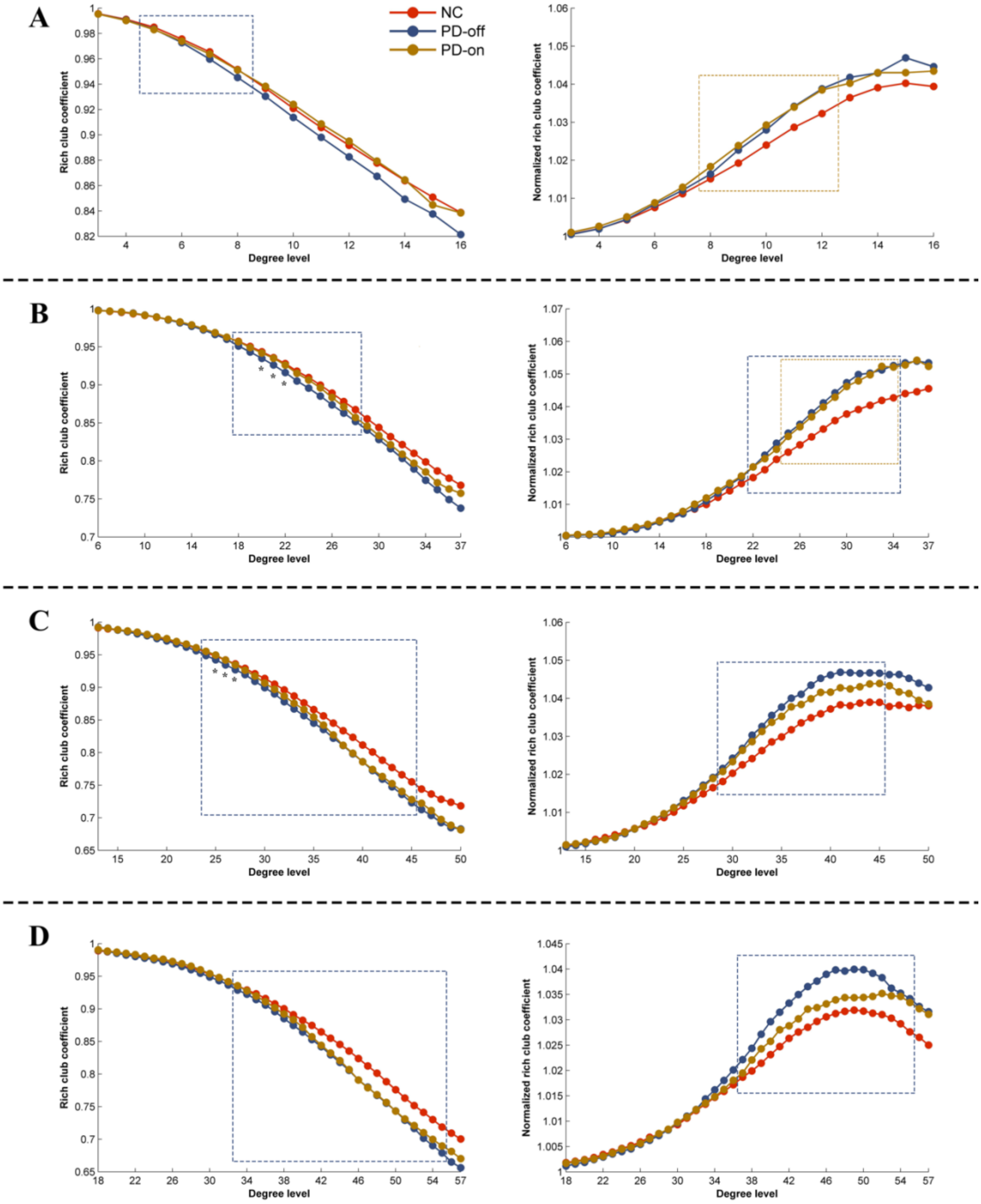

Figure S1 Group-averaged rich club curve of weighted rich-club coefficient (right) and normalized weighted rich-club coefficient (left) for NC (red), PD-off (blue), and PD-on (yellow) across different network sparsity (A = 0.1, B = 0.3, C = 0.4, and D = 0.5). The dash box indicates the differences between NC and PD patients (blue for PD-off, yellow for PD-on) after the FDR correction. * indicate the difference between

PD-off and PD-on with $p < 0.05$. Similar trend of rich-club coefficient and normalized rich-club coefficient was observed across a range of network sparsity.

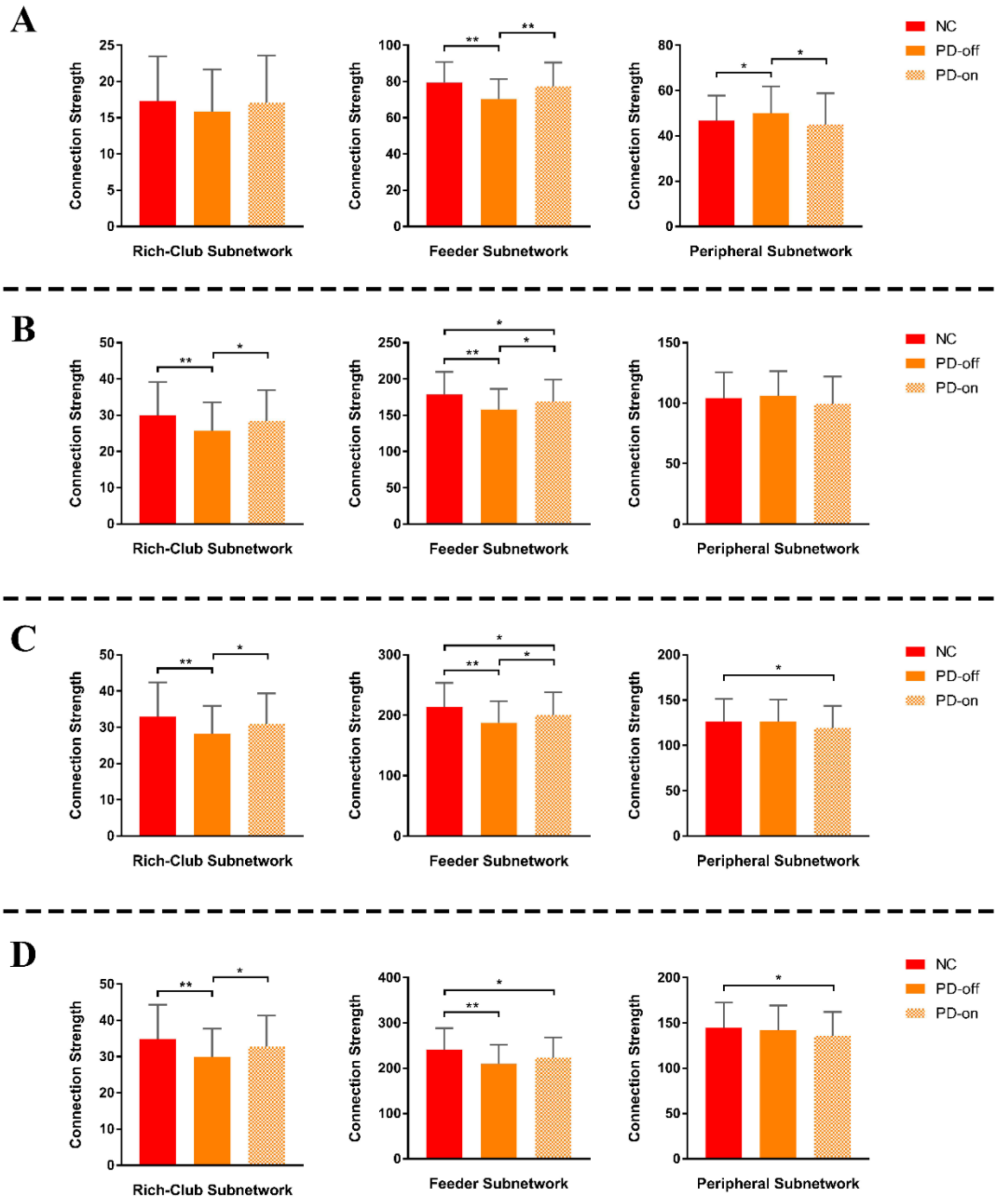

Figure S2 Comparisons of connection strength within three types of subnetworks across different network sparsity (A = 0.1, B = 0.3, C = 0.4, and D = 0.5). **/* indicate the differences corrected by FDR correction/uncorrected $p < 0.05$.

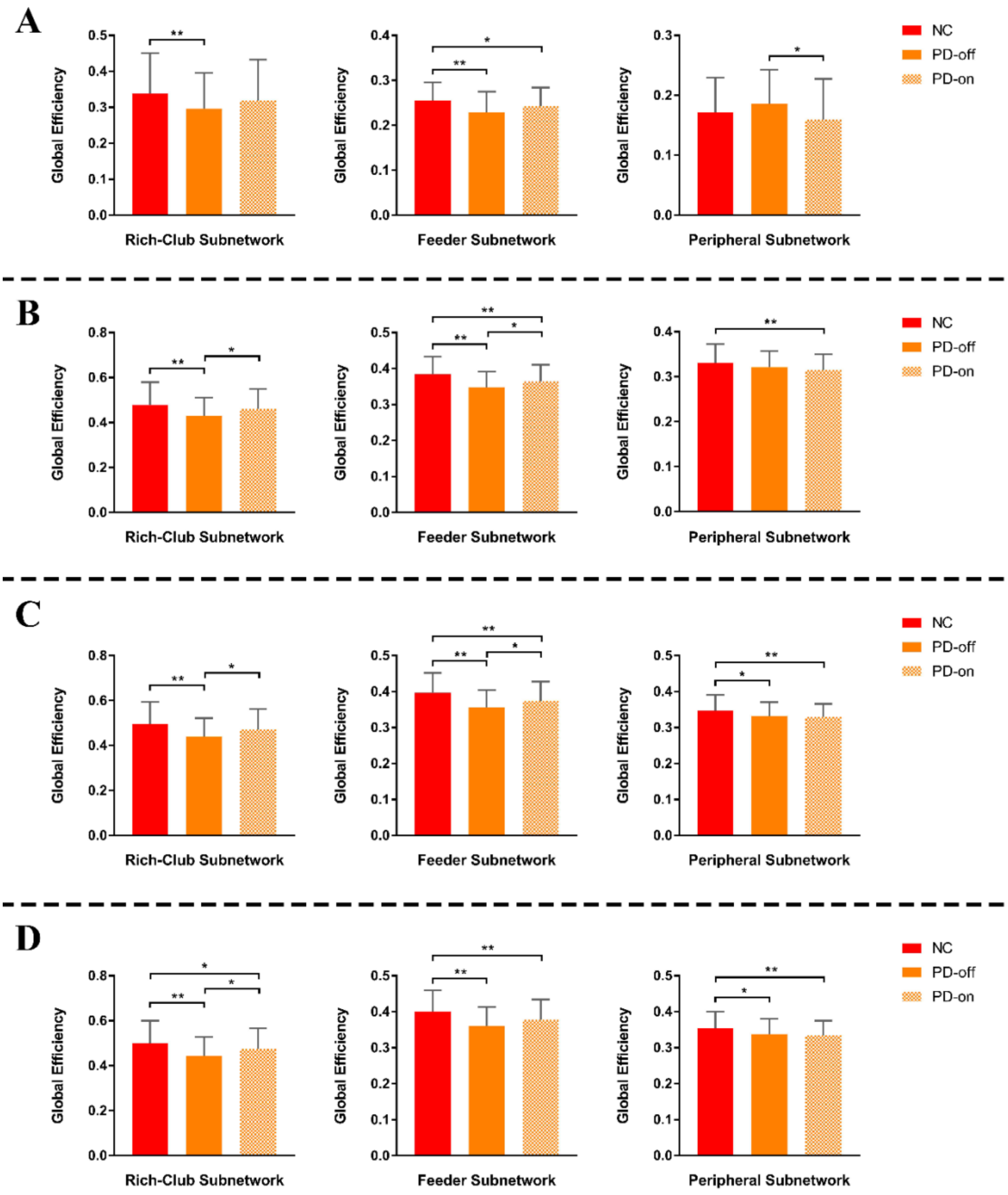

Figure S3 Comparisons of global efficiency within three types of subnetworks across different network sparsity (A = 0.1, B = 0.3, C = 0.4, and D = 0.5). **/* indicate the differences corrected by FDR correction/uncorrected $p < 0.05$.

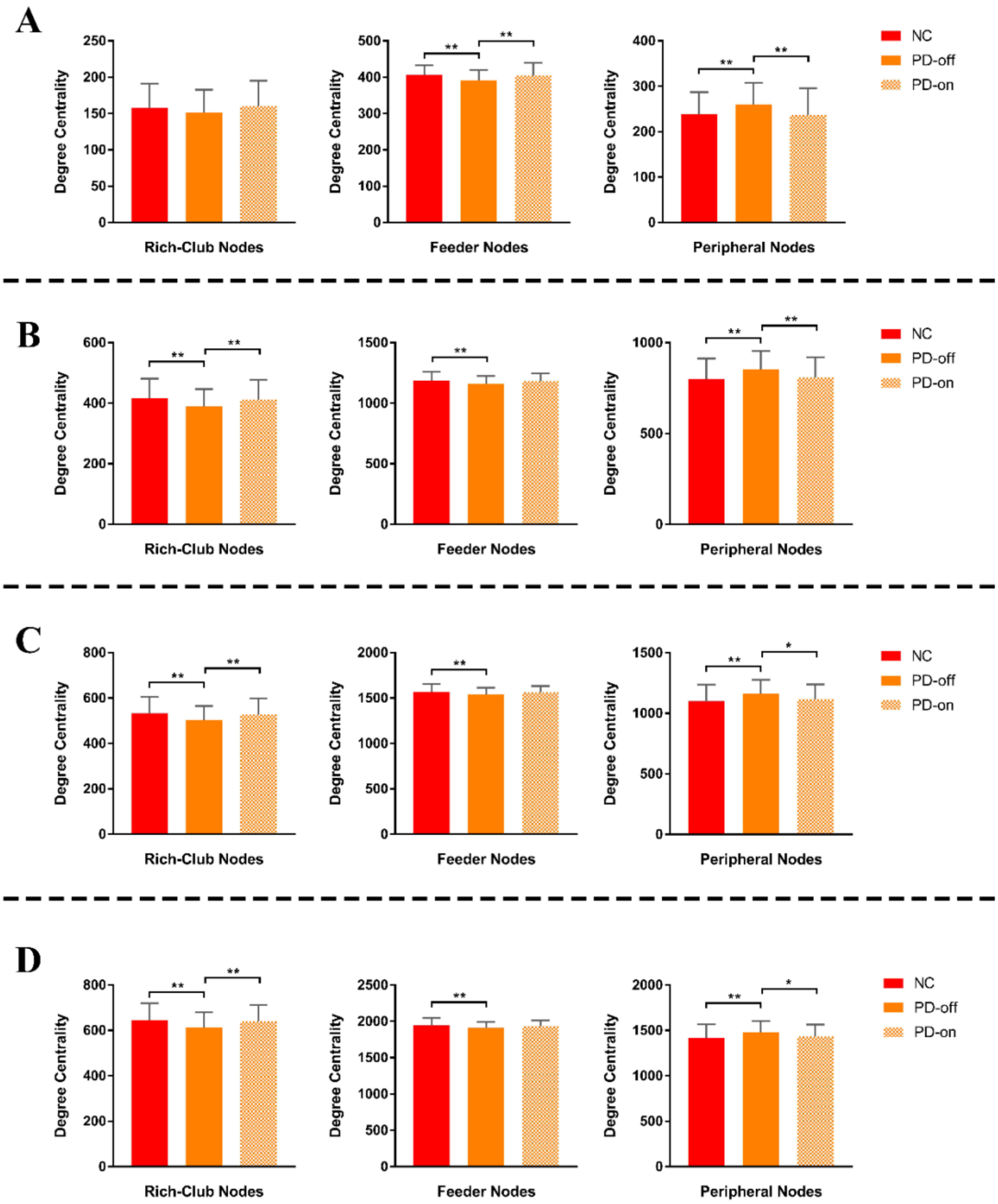

Figure S4 Comparisons of degree centrality for different type of nodes across different network sparsity (A = 0.1, B = 0.3, C = 0.4, and D = 0.5). **/* indicate the differences corrected by FDR correction/uncorrected $p < 0.05$.

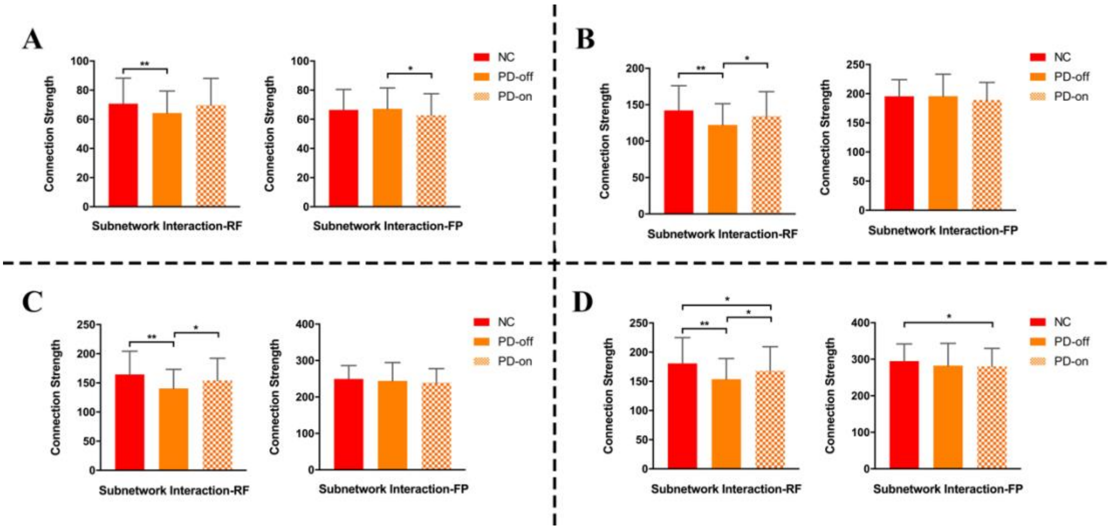

Figure S5 Comparisons of subnetwork interaction between rich-club subnetwork and feeder subnetwork, and between feeder subnetwork and peripheral subnetwork (A = 0.1, B = 0.3, C = 0.4, and D = 0.5). **/* indicate the differences corrected by FDR correction/uncorrected $p < 0.05$.

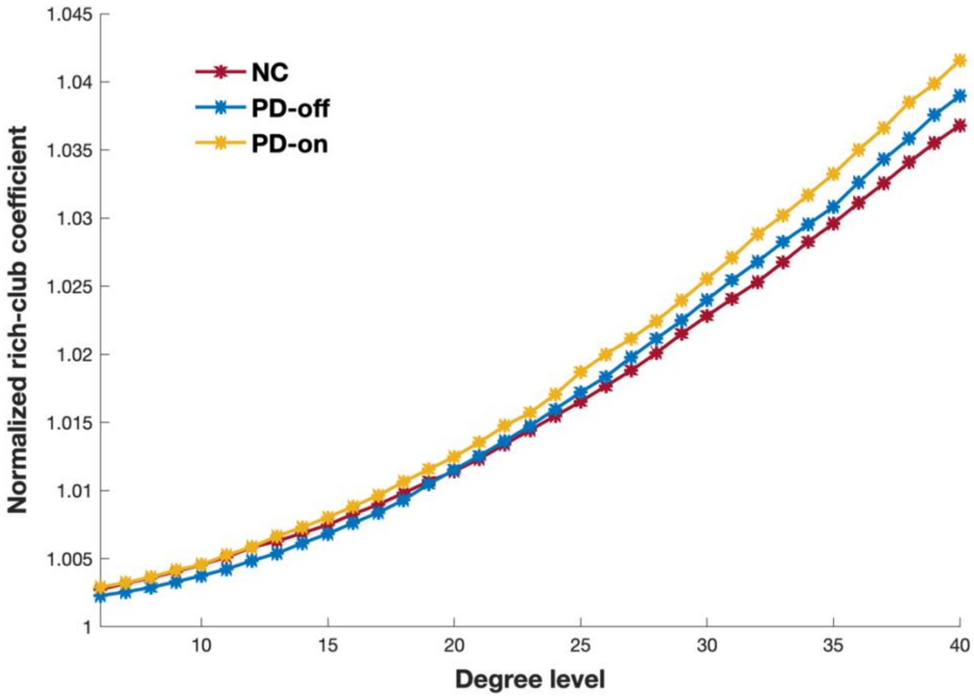

Figure S6 Group-averaged rich club curve of normalized weighted rich-club coefficient for NC, PD-off, and PD-on using Schaefer2018_200 parcel atlas. Both normal control group and PD patients either in OFF- or ON-medication status showed a rich-club organization in their functional network, as the normalized rich-club coefficient higher than 1 across a range of degree.

Reference

1. van den Heuvel MP, Sporns O. Rich-club organization of the human connectome. J Neurosci 2011;31(44):15775-15786.

2. Rubinov M, Sporns O. Complex network measures of brain connectivity: uses and interpretations. Neuroimage 2010;52(3):1059-1069.
